# Efficacy of a stepped-care approach for school-age children with mild to moderate ADHD (ESCAschool-moderate): an adaptive intervention study including a randomized controlled trial

**DOI:** 10.1186/s13034-026-01115-3

**Published:** 2026-07-23

**Authors:** Manfred Döpfner, Christina Dose, Christopher Hautmann, Martin Hellmich, Lea Teresa Kohl, Anne-Katrin Treier, Elena von Wirth, Katja Becker, Daniel Brandeis, Ute Dürrwächter, Julia Geissler, Sarah Hohmann, Martin Holtmann, Michael Huss, Thomas Jans, Anna Kaiser, Johanna Ketter, Tanja Legenbauer, Sabina Millenet, Luise Poustka, Tobias J. Renner, Jasmin Wenning, the ESCAlife Consortium, Nina Christmann, Brigitta Gehrig, Monja Groh, Christine Igel, Karina Janson, Marie-Therese Steiner, Matthias Winkler, Mirjam Ziegler, Nicole Grau, Wiebke Haberhausen, Alisa Hiery, Katja John, Daria Kasperzack, Jennifer Krämer, Christopher Mann, Tanja Mingebach, Anna Maria Paulus, Bastian Schrott, Linda Weber, Anne-Kathrin Ohlsen, Julia Kellner, Katrin Krugmann, Karen Schulze-Husmann, Ann-Katrin Thöne, Paula Vetter, Marcel Romanos, Tobias Johann Renner, Ute Dürrwächer, Lena Flik, Melinda Mross, Anja Pascher, Natalie Richardt, Priska Schneider, Ida Steinacker, Inken Kirschbaum-Lesch, Franziska Martin, Michael Kölch, Stefanie Bienioschek, Henrik Uebel von Sandersleben, Fatmeh Al-Deb’i, Kerstin Konrad, Vanessa Reindl, Jochen Seitz, Tamara Novak, Miriam Remy, Christine Margarete Freitag, Nadine Friedrich, Michael Rösler, Steffen Barra, Petra Retz-Junginger, Birgit Leipnitz, Florence Philipp, Toivo Zinnow, Esther Sobanski, Barbara Alm, Melanie Bleich, Oliver Hennig, Peter Praus, Barbara Scharnholz, Andreas Jochen Fallgatter, Julia Becker-Sadzio, Saskia Deppermann, Thomas Ethofer, Lydia Weber, Michael Erb, Alexandra Phillipsen, Marcel Schulze, Behrem Aslan, Meike Lingen, Maria Steffens, Sina Klüver, Samira Groß, Vivien Groß, Johannes Thome, Beate Krecklow, Wolfgang Retz, Lea Bensing, Sergiy Davydenko, Priscilla Gregório-Hertz, Daniel Turner, Tobias Banaschewski

**Affiliations:** 1 Department of Child and Adolescent Psychiatry, Psychosomatics and Psychotherapy, Faculty of Medicine and University Hospital Cologne, University of Cologne, Cologne, Germany; 2 Center for Child and Adolescent Cognitive Behavior Therapy (CEKIP), Faculty of Medicine and University Hospital Cologne, University of Cologne, Pohligstr. 9, 50969 Cologne, Germany; 3 Department of Medical Statistics, University Medical Center Göttingen, Göttingen, Germany; 4 Clinical Child and Adolescent Psychology and Psychotherapy, Trier University, Trier, Germany; 5 Department of Child and Adolescent Psychiatry, Psychosomatics and Psychotherapy, University of Marburg and University Hospital Marburg (UKGM), Marburg, Germany; 6 Department of Child and Adolescent Psychiatry and Psychotherapy, Central Institute of Mental Health, Medical Faculty, Mannheim/Heidelberg University, Mannheim, Germany; 7 Department of Child and Adolescent Psychiatry and Psychotherapy, University Hospital of Psychiatry Zurich, University of Zurich, Zurich, Switzerland; 8 Neuroscience Center Zurich, University and ETH Zurich, Zurich, Switzerland; 9 Department of Child and Adolescent Psychiatry, Psychosomatics and Psychotherapy, Center of Mental Health, University Hospital of Psychiatry and Psychotherapy Tübingen, Tübingen, Germany; 10 German Center for Mental Health (DZPG), Partner Site Tübingen, Tübingen, Germany; 11 LEAD Graduate School and Research Network, University of Tübingen, Tübingen, Germany; 12 Centre of Mental Health, Department of Child and Adolescent Psychiatry, Psychosomatics and Psychotherapy, University Hospital Würzburg, Würzburg, Germany; 13 Department of Child and Adolescent Psychiatry, Psychotherapy and Psychosomatics, University Medical Centre Hamburg-Eppendorf, Hamburg, Germany; 14 LWL University Hospital Hamm for Child and Adolescent Psychiatry, Psychotherapy and Psychosomatics, Ruhr University Bochum, Hamm, Germany; 15 Department of Psychiatry and Psychotherapy, University Medical Center Mainz, Mainz, Germany; 16 German Center for Mental Health (DZPG), Partner Site Mannheim-Heidelberg-Ulm, Mannheim, Germany; 17 Department of Child and Adolescent Psychiatry, University Medical Center Göttingen, Göttingen, Germany; 18 Department of Child and Adolescent Psychiatry, Center for Psychosocial Medicine, University Hospital Heidelberg, Heidelberg, Germany; 19 Department of Child and Adolescent Psychiatry, Psychosomatics and Psychotherapy, LVR-University Hospital Essen (AöR), University of Duisburg-Essen, Essen, Germany

**Keywords:** Attention-deficit/hyperactivity disorder (ADHD), Children, Stepped-care design, Telephone-assisted self-help, Behavior therapy

## Abstract

**Background:**

This German multicenter study aimed to analyze the efficacy of telephone-assisted self-help (TASH) and changes during subsequent adaptive treatment in children with mild-to-moderate ADHD.

**Methods:**

Participants were children (6;0–11;11 years) with mild-to-moderate ADHD. Study Step 1 comprised a randomized waitlist-controlled trial on the efficacy of three-month, parent-directed TASH. Depending on their response to TASH, in Step 2, children were assigned to booster TASH (full response), behavior therapy (partial response), or pharmacotherapy plus behavior therapy or counseling (non-response) for six months. The primary outcome was the change in blinded-clinician-rated ADHD symptoms; for subsequent changes (Step 2), we considered semi-blinded ratings. The primary analyses were by intention-to-treat.

**Results:**

Of the 163 included children (77.9% boys), 80 were randomized to TASH and 83 to the waitlist control group. Following TASH, 12 children (8.8%) were classified as full responders, 40 (29.2%) as partial responders, and 85 (62.0%) as non-responders. An analysis of covariance did not yield an effect of TASH on the primary outcome (mean between-group difference = 0.00 ± 0.07, 95% *CI*[− 0.14, 0.13]; *p* = 0.95; *d* = –0.01, 95% *CI*[− 0.37, 0.34]). During Step 2, full responders demonstrated a stable symptom level (piecewise mixed-effects model for repeated measures; *d* = − 0.07, 95% *CI*[− 0.61, 0.47], *p* = 0.80), partial responders a small increase in ADHD symptoms (*d* = 0.29, 95% *CI*[0.00, 0.59], *p* = 0.05), and non-responders a large decrease (*d* = − 1.00, 95% *CI*[− 1.27, − 0.73], *p* < 0.001).

**Conclusions:**

TASH is not effective in reducing blinded-clinician-rated ADHD symptoms in children with mild-to-moderate ADHD. Based on the changes within the response groups during Step 2, hypotheses are generated for adaptive treatment after TASH.

*Trial registration* German Clinical Trials Register (DRKS): DRKS0000897; URL: https://drks.de/search/de/trial/DRKS00008973/details; registered on December 18th 2015.

**Supplementary Information:**

The online version contains supplementary material available at 10.1186/s13034-026-01115-3.

## Background

Systematic reviews and meta-analyses indicate small to moderate effects of behavioral interventions on attention-deficit/hyperactivity disorder (ADHD) core symptoms when rated by informants close to the therapeutic setting (e.g., parents in the case of parent training) [1–4]. Smaller or non-significant effects are found when considering more distal ratings (e.g., teachers in the case of parent training) [1, 4, 5]. Additionally, there is evidence demonstrating effects of behavioral interventions on proximal ratings of conduct problems, positive and negative parenting practices, parenting self-concept, social skills, and academic performance, and on distal ratings of conduct problems and positive and negative parenting [1, 4].

Given frequent barriers to access to face-to-face treatment, including limited local treatment options [6, 7], long waiting periods, or personal barriers like fear of stigmatization, self-help interventions provide a low-threshold, easily accessible treatment alternative [8]. There is meta-analytic evidence to support effects of both online and manual-based parent-directed self-help interventions on parent-rated child externalizing behavior, parenting behavior, parental mood and stress, and parenting efficacy [9–11]. Treatment effects seem to be larger for self-help interventions with additional therapist input (e.g., via telephone) [10, 12].

Moreover, meta-analyses point to the efficacy of various medications in treating ADHD core symptoms [13]. In general, stimulant medications have been found to be more effective than non-stimulant medications, with moderate to large effects on blinded ratings of ADHD core symptoms [13, 14]. While some authors conclude that combined pharmacological and behavioral treatment is superior to both behavior therapy or medication alone [15], others report that behavioral interventions, pharmacological interventions, and their combination are similarly effective [16].

Evidence-based US, UK, and German guidelines generally recommend multimodal, stepwise-intensifying treatment, and provide age-specific and partly symptom level-specific recommendations [17–19]. However, the guidelines differ with respect to the precise treatment recommendations and whether they provide different recommendations according to ADHD symptom severity [20]. Following initial psychoeducation, the German guidelines recommend behavioral interventions (especially parent training) for school-age children with mild ADHD, either pharmacotherapy or behavioral interventions for moderate ADHD, and pharmacotherapy as the initial treatment for severe ADHD [17]. For all groups, the German guidelines recommend to add the respective other treatment if symptoms persist. Additionally, the German guidelines specify that parent training may also be provided as an assisted self-help intervention [17].

To the best of our knowledge, only two studies to date have examined an adaptive stepped-care approach for the treatment of ADHD [21–23]. For instance, Pelham et al. randomized 146 children with ADHD (5–12 years) to receive either initial treatment with behavioral interventions or initial pharmacological treatment [23]. Children with insufficient treatment response were subsequently randomized to secondary interventions, which comprised either an increase of the intensity/dose of the initial treatment or the addition of the respective other treatment modality. The authors found superior outcomes in the group that started with behavioral interventions (fewer observed classroom rule violations, better outcomes on parent- and teacher-rated oppositional behavior, better attendance rates of parent training). Adding pharmacotherapy to initial behavioral interventions was superior to vice versa. However, while the study provided important insights into the general utility of a stepped-care approach and the favorable sequence of interventions, there is still a need for studies that consider symptom severity at the beginning of treatment and incorporate low-threshold interventions like (assisted) self-help.

The present article reports on the results of one study arm of the ESCAschool trial [24]. The aim of this study arm was to analyze the efficacy of a parent-directed telephone-assisted self-help intervention (TASH; Step 1) and to exploratively examine within-group changes during subsequent adaptive treatment (Step 2; observational phase) in school-age children with mild to moderate ADHD. The rationale underlying this stepped-care approach was to initially offer low-threshold TASH to a broad sample and to provide more intensive treatment only to children and families with persisting or residual symptoms and impairment. In clinical practice, such an approach may help to improve access to treatment by freeing up treatment capacities for more severely affected individuals. Moreover, the use of low-threshold TASH could enhance the cost-effectiveness of treatment. The findings for the second study arm, which focused on children with severe ADHD, are going to be presented elsewhere (ESCAschool-severe; Döpfner et al., unpublished).

In the first step, we hypothesized that children whose parents directly received a three-month TASH intervention would show a greater reduction in blinded clinician-rated ADHD symptoms (primary outcome Step 1) than children in a waitlist control group (WCG). For the subsequent observational phase (Step 2; lasting for six months), we expected that (a) the improvement in full responders to TASH during Step 1 would remain stable when receiving booster sessions of TASH (semi-blinded clinician ratings of ADHD symptoms; primary outcome Step 2); (b) partial responders to TASH would show a (further) significant reduction in clinician-rated ADHD symptoms under behavior therapy (BT); and (c) non-responders to TASH would show a significant reduction in clinician-rated ADHD symptoms when receiving pharmacotherapy plus behavior therapy or counseling (MED + BT/Couns.). Notably, given the observational nature of Step 2 and the lack of control groups, the analyses for this step can only be considered exploratory and preliminary.

Additionally, from an exploratory perspective, we considered effects on and changes in ADHD symptoms rated by parents and teachers, symptoms of disruptive behavior disorders (DBD) as well as impairment rated by clinicians, parents, and teachers, and parent-rated quality of life, parenting practices, and parental self-efficacy.

## Methods

### Study design and participants

The multicenter ESCAschool study was conducted at nine university hospitals throughout Germany (Bochum, Cologne, Mannheim, Marburg, Tübingen, Würzburg; three additional centers joined in 2017: Essen, Göttingen, Mainz). The study was approved by the local ethics committees of all participating centers. All participating children provided assent, and their parents and all participating teachers provided written informed consent for participation. The study was registered at the German Clinical Trials Register (identifier: DRKS0000897; registered on 18 December 2015; trial closed; https://drks.de/search/de/trial/DRKS00008973/details). Moreover, details regarding study design and methods have been published in a trial protocol [24].

**Fig. 1:**
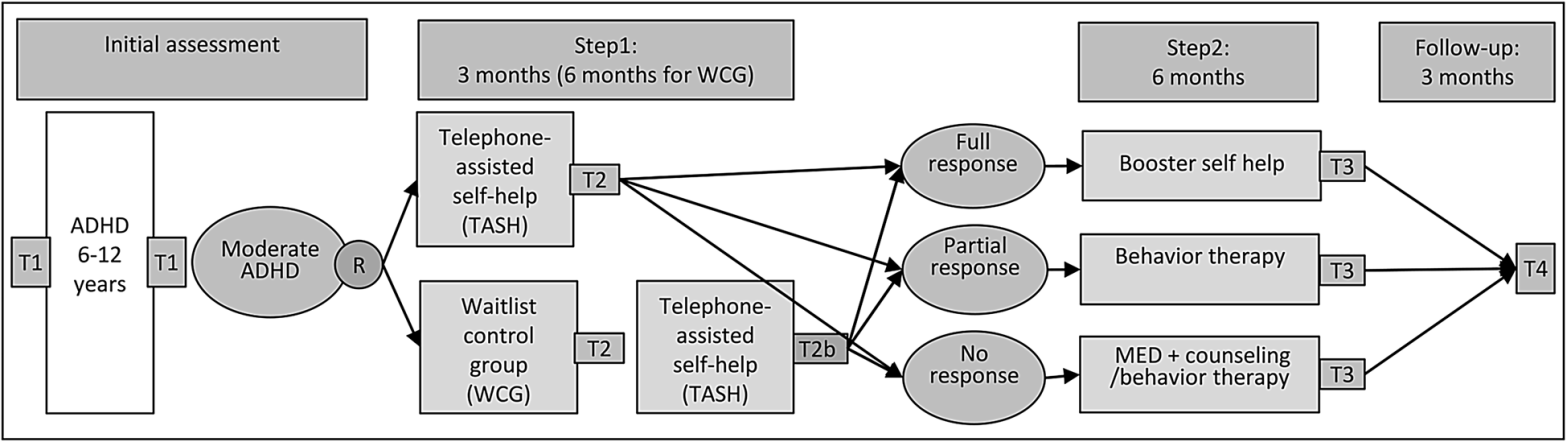
Study design of the mild-to-moderate arm of the ESCAschool study. ADHD = attention-deficit/hyperactivity disorder, T1 = baseline assessment, T2 = three-month assessment point after TASH in the TASH condition or the waiting period in the waitlist control condition, respectively, T2b = six-month assessment point after TASH in participants originally randomized to the WCG, T3 = assessment point after six months of Step 2 treatment, T4 = follow-up assessment point three months after the end of Step 2 treatment, MED = medication

Eligibility criteria and exclusion criteria are displayed in Table 1. During enrollment, children were classified as showing either “severe” or “mild-to-moderate” ADHD symptom levels based on a clinician-administered diagnostic checklist assessing DSM-5 criteria for ADHD (Diagnostic Checklist for ADHD [DCL-ADHD] [25]; semi-blinded ratings) and a corresponding structured interview (ILF-EXTERNAL [26]; see Table 1). Based on this classification, they were assigned to one of two study arms, which represent distinct studies. The present article focuses on the arm for children with mild-to-moderate ADHD. Figure 1 depicts the design for the mild-to-moderate study arm of the ESCAschool study. Deviations from the original study protocol [24] are outlined in the online appendix (Table S1).

**Table 1:** Inclusion and exclusion criteria, severity criteria, and response criteria

Assessment for eligibility	
Inclusion criteria	Age 6;0 to 11;11 years
	School attendance (including special schools)
	Fulfilment of DSM-5 diagnostic criteria for ADHD, assessed with the Diagnostic Checklist for ADHD (DCL-ADHD) based on a semi-structured clinical interview
Exclusion criteria	Child IQ below 80
	Clinical diagnosis of autism spectrum disorder, schizophrenia, bipolar disorder, severe depressive episode, epilepsy, or heart disease
	Insufficient German language or reading skills of the parent primarily involved in treatment
	Current or planned, intensive (i.e., weekly) behavior therapy for child ADHD or oppositional behavior
	Known non-response to all standard ADHD medication (i.e., methylphenidate, dexamphetamine, atomoxetine)
	Psychotropic medication other than for the treatment of ADHD, or neuroleptic medication other than for the treatment of disturbances of impulse control
**Severity criteria (assessment during enrollment; based on semi-blinded clinician ratings)**	
Severe ADHD	Rating ≥ 5 on the semi-blinded clinician-rated CGI severity scale (range 1–7)^a^
	Mean item score ≥ 2 (possible range 0–3) on the inattention scale and/or the hyperactivity/impulsivity scale of the DCL-ADHD
Mild to moderate ADHD	All children meeting the eligibility criteria and not meeting the criteria for “severe ADHD”
**Response criteria (classification after treatment with TASH in Step 1; based on semi-blinded clinician ratings)**	
Full response	Semi-blinded clinician-rated CGI severity scale score < 3
	Mean score ≤ 1 on both the inattention scale and the hyperactivity/impulsivity scale of the DCL-ADHD
Partial response	Mean item score > 1 on the DCL-ADHD inattention scale or a mean score > 1 on the DCL-ADHD hyperactivity/impulsivity scale or a semi-blinded clinician-rated CGI severity scale score ≥ 3
	Reduction in the DCL-ADHD total score by at least 20% compared to baseline
Non-response	Mean item score > 1 on the DCL-ADHD inattention scale or mean score > 1 on the DCL-ADHD hyperactivity/impulsivity scale or a semi-blinded clinician-rated CGI severity scale score ≥ 3
	Reduction in the DCL-ADHD total score of less than 20% compared to baseline

IQ = intelligence quotient, DCL-ADHD = Diagnostic Checklist for ADHD, CGI = Clinical Global Impressions, TASH = telephone-assisted self-help

^a^We considered the pre–post difference on the CGI severity scale, rather than the improvement scale, to assess change during Step 1, as this approach is consistent with the inclusion criteria and represents a more direct measure of change.

Step 1 consisted of a randomized controlled trial (RCT) on the efficacy of a parent-directed TASH intervention. Participating families were randomly assigned to either TASH or a WCG. Parents in the TASH condition (and also the teachers, if both the parents and the teachers agreed) participated in the three-month TASH intervention, while families in the WCG did not receive any study-specific treatment during this period. However, families in the WCG received TASH after the three-month waiting period. After participating in the TASH intervention, children from both groups were classified in terms of their response to this treatment, taking into account current ADHD symptoms and improvement during TASH (for a definition of the response criteria, see Table 1). In Step 2, parents (and optionally also teachers) of children showing a “full response” were offered booster self-help sessions, families of children showing a “partial response” were offered BT, and families of “non-responders” were offered MED + BT/Couns.

The a priori planned overall sample size for the mild-to-moderate study arm was *n* = 144 [24]. Assessments in the families initially randomized to TASH were carried out at baseline (T1), 3 months (interim assessment, T2), 9 months (post-assessment, T3), and 12 months (follow-up assessment, T4); assessments in the families randomized to the WCG were carried out at baseline (T1), 3 months (T2), 6 months (T2b), 12 months (T3), and 15 months (T4; see Fig. 1).

Recruitment was carried out from March 2016 to November 2018 and was mainly achieved through outpatient units of the nine participating study centers. Additionally, written study information was given to schools, local welfare offices, local health departments, pediatricians, child and adolescent psychiatrists, occupational therapists, counseling centers, academic tutoring institutes, and self-help groups, and was provided on the internet (https://www.esca-life.org), and in local newspaper articles.

### Randomization and masking

For Step 1, participating families were randomly assigned to either TASH or the WCG. Both registration for the study and randomization followed a strictly predefined procedure [24]. To ensure concealment, randomization was performed by an independent study center (Clinical Trials Unit Freiburg). Randomization forms were completed at the study centers and sent to the Clinical Trials Unit Freiburg per fax. In the case of implausible or incomplete information in this form, the Clinical Trials Unit returned a query fax to the study center to ask for clarification. To ensure that treatment assignment was unbiased and concealed from patients and staff at the study sites, the randomization code was generated by the Clinical Trials Unit Freiburg using validated programs. Participants were block-randomized with blocks of variable length and an allocation ratio of 1:1. The block lengths were documented separately and not disclosed to the study sites.

Due to the nature of the study interventions, neither the participants, the therapists, nor the study staff involved in the organization of the study could be blinded to the study conditions. However, efforts were made to blind the clinicians who assessed symptoms of ADHD and disruptive behavior disorders (DBD) based on parent interviews to the study conditions and assessment points. Notably, it was not possible to ensure full blinding due to staff meetings or statements made by parents, which is why we consider the respective clinician ratings as “semi-blinded”. For the analysis of the RCT in Step 1, we additionally considered “fully blinded” clinician ratings of ADHD and DBD symptoms. These ratings were based on video or audio recordings of parent interviews conducted by the semi-blinded clinician, with all references to the treatment condition or the time of assessment being deleted.

### Interventions

The TASH parent program consists of eight self-help booklets on ADHD and behavior modification techniques, including contingency management [27], as well as ten accompanying, approximately weekly telephone consultations of about 20 to 30 min. The TASH teacher program comprises four booklets and four telephone consultations, each lasting up to 60 min. The child’s teacher only participated in the TASH intervention if both the parents and the teacher consented to the teacher’s participation. For the contents of the parent- and teacher-directed booklets, see Table S2.

In the case of full response to TASH, parents received two TASH booster sessions and teachers optionally received one TASH booster session in Step 2. Participants classified as partial responders received BT in Step 2, which encompassed 20 weekly sessions and comprised parent management training, child-focused interventions (especially organizational skills training) and, optionally, teacher-focused interventions (see Table S3). The interventions were primarily based on the German “Therapy Program for Children with Hyperactive and Oppositional Problem Behaviors” [28].

Non-responders to TASH were advised to consult a study center physician or a local physician to assess the indication for pharmacological treatment at the beginning of Step 2. Besides asking the physicians to adhere to current treatment guidelines, there was no study-related specification for pharmacological treatment. The number of physician visits for the provision of pharmacotherapy depended on the individual needs of the children and their families (with an expected number of approximately four visits). Additionally, non-responders participated in either 20 sessions of BT (see Table S3) or 6 sessions of counseling (see Table S4). Originally, all non-responders to TASH were to receive pharmacotherapy (if indicated) in combination with BT in the second study step. However, due to an unexpectedly high number of non-responders and limited resources at some participating centers, treatment options were expanded to include BT or counseling. The specific study treatment offered (BT or counseling) was determined based on the needs of the families as well as the usual practice and the resources available at the respective study site. Counseling and BT were based on the same manuals and materials and comprised the same key components; counseling essentially represented a less intensive version of BT. Only a small number of children received counseling instead of BT. Conversely, some families attended only a limited number of BT sessions (see Results section), rendering the BT and counselling interventions delivered even more similar. On the other hand, the large variability in the number of completed sessions and the flexibility regarding medication use resulted in heterogeneous treatment in this group. Although close to clinical reality, it should be noted that the findings for this condition cannot be attributed with certainty to changes during a specific treatment.

Both TASH and BT were provided by child and adolescent psychotherapists and by educationalists or psychologists in training to become child and adolescent psychotherapists. Various measures were taken to ensure treatment fidelity. First, all therapists providing the TASH intervention, BT, and/or counseling were trained in manualized treatment procedures. Second, after each session, the therapists completed a structured protocol assessing treatment integrity. Third, the TASH, BT, and counselling sessions were regularly supervised by senior supervisors, either in person or by telephone. For BT, an individualized treatment plan was developed for each participating child with the support of a senior supervisor after the fifth session. For the supervision of the TASH intervention, the telephone counseling sessions were recorded in audio files. For the supervision of BT, video recordings of at least two sessions were considered.

### Outcomes

The primary outcome in Step 1 was the change in ADHD symptoms as rated by a blinded clinician using the DCL-ADHD (18 items); the primary outcome for the within-group analyses of changes during Step 1 and Step 2 was the change in semi-blinded clinician-rated ADHD symptoms. We chose the blinded clinician-rated DCL-ADHD as the primary outcome measure given that it is a widely used measure for the assessment of ADHD symptoms in Germany, has good psychometric properties [26], and is recommended in the German ADHD guidelines [17]. Moreover, we decided to use blinded clinical ratings based on parent interviews in order to counter common problems associated with blinding in studies on psychosocial interventions. In previous literature, ratings by persons distal to the therapeutic setting (e.g., teacher ratings on effects of parent training or vice versa) are often considered as “probably blinded” ratings [1, 4, 5]. However, the reliability and ecological validity of these ratings is questionable, as behavioral treatments for ADHD have rather domain-specific effects [2, 3]. By using clinician ratings based on parent information, we were able to ensure blinding while gathering information less distal to the therapeutic setting.

Secondary outcomes comprised semi-blinded-clinician-, parent-, and teacher-rated ADHD symptoms, clinician-, parent-, and teacher-rated DBD symptoms and ADHD- and DBD-related impairment, as well as parent-rated child internalizing symptoms, functional impairment, quality of life, positive and negative parenting practices, and parental self-efficacy (in terms of being able to deal with the child’s problem behaviors). For the measures used to assess the primary and secondary outcomes and their psychometric properties, see Table S5. Parental satisfaction with Step 1 treatment was assessed using eight self-developed items at T2, and satisfaction with Step 2 treatment was measured at T3. Psychological side effects were assessed using a predefined checklist during the semi-blinded clinical interview conducted with the parent after each treatment step. The checklist comprised five items: lack of leisure time and limited contact with peers; increased conflicts with family members or peers; fear that others might find out about the therapy; feeling of change for the negative; and irritability or bad mood. In addition, an open-ended question was included. Responses to the open question that reflected one of the predefined categories were assigned accordingly. The remaining responses primarily referred to time or organizational burden; these were introduced as an additional category for reporting purposes. Responses not fitting into any of the categories were classified as “other.” During the interview, parents reported psychological side effects both for themselves and for their child.

### Statistical analyses

The analyses were conducted using the software SPSS Statistics (version 29, IBM Corp., Armonk, NY, USA) and R Statistics (version 2024.04.2) [29]. The primary analysis of the primary and secondary outcomes was by intention-to-treat (ITT). The full analysis sample (FAS) for the analyses of the RCT outcomes in Step 1 comprised all children who had been randomized (TASH: *n* = 80, WCG: *n* = 83). For the ITT analyses of within-group changes in the different response groups during the course of the study, the FAS comprised all children who were assigned to one of the treatment arms in Step 2. We additionally performed per-protocol (PP) analyses in children who had received a minimum number of sessions (Step 1/TASH: ≥ 7 telephone counselling sessions with the parents; Step 2/BT: ≥ 14 sessions) and for whom no major protocol violations were recorded (e.g., false classification of response to TASH, see Table S1).

We tested for group differences in the primary and secondary outcomes of the RCT in Step 1 by performing univariate analyses of covariance (ANCOVAs), which were based on linear regression analyses. The change between T1 and T2 for a respective outcome variable was considered as the dependent variable, study condition was considered as the independent variable, and study site and baseline scores of the respective outcome variables were entered into the models as covariates. To account for the different enrollment numbers of the study centers, we computed unbalanced estimated marginal means from the model equations.

Missing values were not replaced for conducting the primary analyses for Step 1. Thus, the regression analyses considered only cases with available data at both T1 and T2 (primary outcome, blinded clinician-rated ADHD symptoms: TASH: *n* = 59, WCG: *n* = 58). However, we additionally performed sensitivity analyses on all randomized cases, with missing values imputed using Bayesian Stochastic regression [30]. No values were imputed for the teacher-rated variables due to a high percentage of missing values (> 50%); the respective scales were thus excluded from the sensitivity analyses.

Regarding changes within the different response groups (full, partial, and non-responders to TASH) over the entire course of the study (exploratory, hypothesis-generating analyses), we analyzed piecewise mixed-effects models for repeated measures [31], using a heterogeneous first-order autoregressive covariance structure for the repeated measurements. The models included fixed effects for the changes during Step 1, Step 2, and the three-month follow-up period (from T3 to T4), with study site as a covariate. These models considered all cases allocated to one of the Step 2 treatments. For these cases, at least the assessment at the beginning of Step 2 (“Step 2 baseline”) was available. PP analyses regarding changes over the entire course of study were only performed for partial responders to TASH, who received BT in Step 2, as only a small number of major protocol violations were reported for participants in the other two response groups (full responders, non-responders; see Table S1).

For both between-group differences (RCT Step 1) and within-group differences (Step 1, Step 2, and follow-up period), Cohen’s d was considered as a measure of effect size [32]. As we used clearly predefined primary and secondary outcome variables, we did not control for multiple testing [33, 34].

## Results

### Participant flow and sample characteristics at baseline

**Fig. 2:**
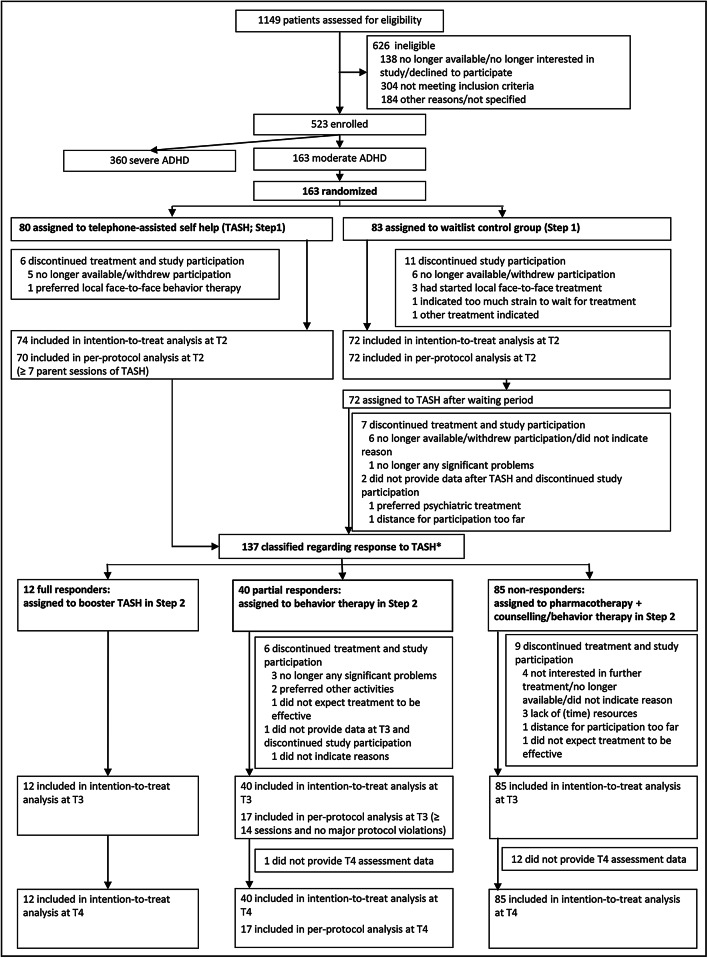
Participant flow. ADHD = attention-deficit/hyperactivity disorder, T2 = three-month assessment point after TASH (TASH condition) or after waitlist period (waitlist control condition), T3 = assessment point after six months of Step 2 treatment, T4 = follow-up assessment point three months after the end of Step 2 treatment. The sample sizes for the analyses refer to unblinded clinician-rated ADHD symptoms, as this is the measure with the highest number of cases per analysis. * Six participants were falsely classified as partial responders but were actually low responders. Five participants were falsely classified as low responders but were actually partial responders. These participants were excluded from the per-protocol analyses.

Figure 2 displays the participant flow. Of the 523 children included in the study, 163 were assigned to the mild-to-moderate study arm. In Step 1, 80 children were randomized to TASH and 83 children were randomized to the WCG. The children’s mean age was 8.86 years (*SD* = 1.43) and 127 (77.9%) were boys. For further demographic and baseline clinical characteristics, see Tables S6 and S7. At T1, two children randomized to TASH and six children randomized to the WCG received ADHD medication (χ^2^ = 1.95, *df* = 1, *p* = 0.28), with no significant group difference in the dosage of methylphenidate, which was the most frequently used substance (see Table S8).

After completing TASH, 12 children (8.8%) were classified as full responders and received TASH booster sessions in Step 2; 40 children (29.2%) were classified as partial responders and were assigned to BT in Step 2; and 85 children (62.0%) were classified as non-responders and received MED + BT/Couns. in Step 2. Due to calculation errors, five partial responders were misclassified as non-responders, and six non-responders were misclassified as partial responders. These children were included in the ITT sample for the analyses including Step 2 and analyzed according to their allocated arm, but were excluded from the PP sample.

### Treatment characteristics

On average, parents randomized to TASH in Step 1 participated in 8.70 (*SD* = 2.85, range 0–10) telephone counseling sessions (teachers: *M* = 1.91, *SD* = 1.85, range 0–4; 46 teachers participated) and parents receiving TASH after the waiting period received an average of 8.89 (*SD* = 4.86, range 0–10) sessions (teachers: *M* = 1.34, *SD* = 1.68, range 0–4; 38 teachers participated). Parents of ten children in the TASH condition participated in fewer than seven telephone consultations, which was considered a protocol violation (see Table S1). Data from these families were excluded from the PP analyses. During Step 2, full responders to TASH received an average of 1.83 (*SD* = 0.39; range 1–2) booster sessions (teachers: *M* = 0.33, *SD* = 0.99, range 0–1), partial responders to TASH received an average of 11.58 (*SD* = 6.79, range 0–20) sessions of BT, and non-responders to TASH received an average of 8.06 (*SD* = 8.26, range 0–20) sessions of BT/Couns.. Among the partial responders, 16 children participated in fewer than 14 sessions; their data were excluded from the PP analyses. In the group of non-responders, treatment was quite heterogeneous: 15 children received medication alone, eight children received behavioral treatment alone (minimum one session), 17 children received combined treatment, and eight children received no treatment at all. For a further 22 children who received BT/counseling, we cannot state with any certainty whether or not they received medication due to a large amount of missing data on pharmacological treatment. Finally, for 15 children who did not participate in BT/Couns., data on pharmacological treatment were missing. Of the 48 participants with available data on pharmacological treatment, 32 were prescribed medication. When considering the cases with missing data, the possible range of children receiving medication lay between 32 and 69 (38 to 81%). Given the different treatments, varying treatment intensity, and the large proportion of missing data on medication status in this condition, no inferences can be made regarding a specific, predefined intervention; rather, the findings pertain to heterogeneous intensified treatments in non-responders to TASH.

There was no significant difference between the RCT conditions regarding the utilization of additional, non-study-related treatments, including medication (see Tables S8 and S9). Two of the full responders to TASH and three of the partial responders began pharmacotherapy during Step 1. For these participants, we cannot determine whether their response was attributable to TASH, their pharmacological treatment, or both. One child started pharmacotherapy during Step 2 treatment with BT (see Table S8).

### RCT step 1: effects of TASH

An ITT ANCOVA did not yield a significant group difference in blinded clinician-rated ADHD symptoms (primary outcome). However, the analyses revealed significant group differences in semi-blinded clinician-rated ADHD-inattentive symptoms, overall ADHD symptoms, and ADHD-related impairment. Notably, these semi-blinded ratings might be associated with more bias than the blinded ones. Moreover, the analyses yielded significant group differences in parent-rated DBD-related impairment and negative parenting behaviors. The associated between-group effect sizes were small to moderate (see Table 2; for descriptive statistics, see Table S7). Moreover, at T2, there was a significant difference in both the Clinical Global Impression (CGI) [35] severity score (*Mdn*_TASH_ = 4 [*IQR* = 1], *Mdn*_WCG_ = 4 [*IQR* = 1], *U* = 1902.50, *p* = 0.002) and the CGI improvement score (*Mdn*_TASH_ = 3 [*IQR* = 1], *Mdn*_WCG_ = 4 [*IQR* = 1], *U* = 1378.50, *p* < 0.001) in favor of TASH.

**Table 2:** Results of the ANCOVAs for the group comparisons regarding the mean change from baseline (T1) to 3 months (T2): intention-to-treat analysis

	TASH			WCG			TASH vs. WCG			
	*n*	Estimated marginal mean ∆T1− T2 (95% CI)	*d*_intra_ (95% CI)	*n*	Estimated marginal mean ∆T1− T2 (95% CI)	*d*_intra_ (95% CI)	Difference between estimated marginal means (95% CI)	*SE*	*p*	*d*_inter_ (95% CI)
Blinded clinician										
DCL-ADHD Total	59	− 0.13	− 0.33	58	− 0.12	− 0.32	0.00	0.07	0.95	− 0.01
		(− 0.22, − 0.03)	(− 0.58, − 0.08)		(− 0.22, − 0.03)	(− 0.57, − 0.07)	(− 0.14, 0.13)			(− 0.37, 0.34)
DCL-ADHD Impairment	55	− 0.09	− 0.19	49	− 0.01	− 0.02	− 0.08	0.08	0.29	− 0.17
		(− 0.20, 0.01)	(− 0.40, 0.02)		(− 0.12, 0.10)	(− 0.25, 0.20)	(− 0.23, 0.07)			(− 0.48, 0.15)
DCL-DBD Total	56	− 0.10	− 0.32	49	− 0.03	− 0.10	− 0.07	0.05	0.16	− 0.21
		(− 0.16, − 0.04)	(− 0.51, − 0.12)		(− 0.10, 0.03)	(− 0.32, 0.10)	(− 0.16, 0.03)			(− 0.50, 0.08)
DCL-DBD ODD	56	− 0.17	− 0.33	49	− 0.09	− 0.18	− 0.08	0.08	0.37	− 0.15
		(− 0.28, − 0.06)	(− 0.55, − 0.11)		(− 0.22, 0.03)	(− 0.42, 0.05)	(− 0.24, 0.09)			(− 0.47, 0.18)
DCL-DBD Impairment	31	− 0.06	− 0.12	27	− 0.04	− 0.07	− 0.03	0.13	0.84	− 0.05
		(− 0.24, 0.11)	(− 0.45, 0.21)		(− 0.22, 0.15)	(− 0.42, 0.28)	(− 0.29, 0.23)			(− 0.54, 0.44)
Semi-blinded clinician										
DCL-ADHD Total	74	− 0.15	− 0.47	72	− 0.02	− 0.05	− 0.14	0.07	0.045	− 0.42
		(− 0.25, − 0.06)	(− 0.77, − 0.18)		(− 0.11, 0.08)	(− 0.34, 0.24)	(− 0.27, 0.00)			(− 0.84, − 0.01)
DCL-ADHD Impairment	73	− 0.18	− 0.40	72	− 0.03	− 0.07	− 0.15	0.07	0.049	− 0.33
		(− 0.28. − 0.08)	(− 0.63, − 0.17)		(− 0.13, 0.07)	(− 0.30, 0.16)	(− 0.29, 0.00)			(− 0.66, 0.00)
DCL-DBD Total	74	− 0.10	− 0.30	70	− 0.06	− 0.18	− 0.04	0.04	0.30	− 0.12
		(− 0.16, − 0.05)	(− 0.46, − 0.14)		(− 0.12, 0.00)	(− 0.34, − 0.01)	(− 0.12, 0.04)			(− 0.36, 0.11)
DCL-DBD ODD	74	− 0.14	− 0.27	70	− 0.08	− 0.16	− 0.06	0.07	0.39	− 0.12
		(− 0.24, − 0.05)	(− 0.46, − 0.09)		(− 0.18, 0.02)	(− 0.35, 0.03)	(− 0.20, 0.08)			(− 0.38, 0.15)
DCL-DBD Impairment	73	− 0.07	− 0.12	71	0.07	0.12	− 0.15	0.09	0.09	− 0.24
		(− 0.19, 0.05)	(− 0.31, 0.08)		(− 0.05, 0.20)	(− 0.08, 0.32)	(− 0.32, 0.02)			(− 0.52, 0.04)
Parents										
SCL-ADHD Total	68	− 0.11	− 0.22	68	− 0.04	− 0.07	− 0.08	0.07	0.27	− 0.15
		(− 0.21, − 0.02)	(− 0.41, − 0.03)		(− 0.13, 0.06)	(− 0.26, 0.12)	(− 0.22, 0.06)			(− 0.42, 0.12)
SCL-ADHD Impairment	62	− 0.07	− 0.10	62	0.02	0.03	− 0.09	0.08	0.29	− 0.13
		(− 0.18, 0.04)	(− 0.28, 0.07)		(− 0.10, 0.13)	(− 0.15, 0.20)	(− 0.25, 0.08)			(− 0.38, 0.11)
SCL-DBD Total	68	− 0.07	− 0.16	69	− 0.02	− 0.05	− 0.05	0.05	0.32	− 0.11
		(− 0.14, 0.00)	(− 0.31, − 0.01)		(− 0.09, 0.04)	(− 0.20, 0.10)	(− 0.15, 0.05)			(− 0.32, 0.11)
SCL-DBD ODD	68	− 0.07	− 0.11	69	0.01	0.01	− 0.08	0.08	0.31	− 0.12
		(− 0.19, 0.04)	(− 0.27, 0.06)		(− 0.10, 0.12)	(− 0.15, 0.18)	(− 0.24, 0.08)			(− 0.36, 0.11)
SCL-DBD Impairment	63	− 0.13	− 0.17	60	0.15	0.20	− 0.28	0.10	0.008	− 0.37
		(− 0.26, 0.01)	(− 0.35, 0.02)		(0.01, 0.29)	(0.01, 0.39)	(− 0.48, − 0.07)			(− 0.64, − 0.10)
CBCL Internalizing	68	0.20	0.03	70	0.48	0.08	− 0.28	0.83	0.74	− 0.05
		(− 0.97, 1.36)	(− 0.16, 0.23)		(− 0.67, 1.63)	(− 0.11, 0.27)	(− 1.92, 1.36)			(− 0.32, 0.23)
WFIRS Functional Impairment	67	− 0.06	− 0.14	69	0.03	0.06	− 0.09	0.05	0.06	− 0.20
		(− 0.13, 0.00)	(− 0.29, 0.01)		(− 0.04, 0.09)	(− 0.09, 0.21)	(− 0.18, 0.01)			(− 0.41, 0.01)
KIDSCREEN Quality of Life	66	− 0.16	− 0.03	65	− 0.17	− 0.03	0.01	0.62	0.99	0.00
		(− 1.02, 0.70)	(− 0.21, 0.14)		(− 1.04, 0.70)	(− 0.21, 0.14)	(− 1.22, 1.23)			(− 0.25, 0.25)
FPNE Positive Parenting	68	0.05	0.14	70	− 0.01	− 0.03	0.06	0.04	0.12	0.17
		(− 0.01, 0.11)	(− 0.01, 0.30)		(− 0.07, 0.04)	(− 0.18, 0.12)	(− 0.02, 0.14)			(− 0.05, 0.39)
FPNE Negative Parenting	67	− 0.14	− 0.38	69	0.02	0.05	− 0.15	0.05	0.001	− 0.43
		(− 0.20, − 0.07)	(− 0.55, − 0.20)		(− 0.04, 0.08)	(− 0.12, 0.22)	(− 0.24, − 0.06)			(− 0.67, − 0.18)
PSBC Parental Self-efficacy	66	0.14	0.29	68	0.05	0.11	0.09	0.06	0.15	0.18
		(0.06, 0.22)	(0.12, 0.47)		(− 0.03, 0.13)	(− 0.06, 0.28)	(− 0.03, 0.20)			(− 0.06, 0.43)
Teachers										
SCL-ADHD Total	33	− 0.19	− 0.34	37	− 0.03	− 0.06	− 0.16	0.10	0.13	− 0.28
		(− 0.34, − 0.04)	(− 0.59, − 0.08)		(− 0.17, 0.11)	(− 0.30, 0.19)	(− 0.36, 0.05)			(− 0.64, 0.08)
SCL-ADHD Impairment	27	− 0.16	− 0.24	29	− 0.03	− 0.04	− 0.14	0.15	0.37	− 0.20
		(− 0.38, 0.05)	(− 0.54, 0.07)		(− 0.23, 0.18)	(− 0.33, 0.25)	(− 0.44, 0.16)			(− 0.63, 0.24)
SCL-DBD Total	35	− 0.15	− 0.26	37	− 0.02	− 0.03	− 0.13	0.09	0.14	− 0.23
		(− 0.27, − 0.03)	(− 0.47, − 0.05)		(− 0.14, 0.10)	(− 0.24, 0.17)	(− 0.31, 0.04)			(− 0.53, 0.07)
SCL-DBD ODD	35	− 0.16	− 0.22	37	0.06	0.08	− 0.22	0.12	0.06	− 0.30
		(− 0.33, 0.00)	(− 0.44, 0.00)		(− 0.10, 0.21)	(− 0.14, 0.29)	(− 0.45, 0.01)			(− 0.61, 0.01)
SCL-DBD Impairment	23	− 0.27	− 0.29	28	− 0.07	− 0.08	− 0.20	0.17	0.26	− 0.21
		(− 0.52, − 0.02)	(− 0.57, − 0.02)		(− 0.30, 0.15)	(− 0.33, 0.17)	(− 0.54, 0.15)			(− 0.59, 0.16)

The ANCOVAs controlled for baseline scores on the respective outcome measure and for study site. TASH = telephone-assisted self-help, WCG = waitlist control group, DCL-ADHD = Diagnostic Checklist for Attention-Deficit/Hyperactivity Disorder, ODD = Oppositional Defiant Disorder, SCL-ADHD = Symptom Checklist for Attention-Deficit/Hyperactivity Disorder, SCL-DBD = Symptom Checklist for Disruptive Behavior Disorders, CBCL = Child Behavior Checklist, WFIRS = Weiss Functional Impairment Rating Scale, KIDSCREEN = German questionnaire for the assessment of health-related quality of life, FPNE = German questionnaire for the assessment of parenting behaviors, PSBC = German version of the Problem Setting and Behavior Checklist for the assessment of parental self-efficacy

CI = confidence interval, ∆T1-T2 = change from baseline to three-month assessment time point, *p* = significance value, *d* = Cohen’s d (effect size), *SE* = standard error

Notably, there was a relatively high proportion of missing values in most ITT analyses, which limited the interpretability of the findings (blinded clinician ratings: 26.25% to 61.25% in TASH and 30.12% to 67.47% in the WCG; semi-blinded clinician ratings: 7.50% to 8.75% in TASH and 13.25% to 15.66% in the WCG; parent ratings: 15.00% to 22.50% in TASH and 15.66% to 27.71% in the WCG; for sample sizes in the ITT analyses, see Table 2). Therefore, we additionally performed sensitivity analyses based on imputed data, which yielded results similar to those of the original ITT analyses. Besides the effects already found in the analyses based on the original data, these analyses yielded additional effects on semi-blinded clinician-rated DBD-related impairment and on parent-rated functional impairment (see Table S10). For the teacher ratings, we did not compute analyses based on imputed data due to the high proportion of missing values (TASH: 56.25% to 71.25%; WCG: 55.42% to 66.27%). Consequently, the results for the teacher ratings should be interpreted with caution and can only be regarded as preliminary.

In general, the ANCOVAs in the PP sample (based on the original data, without imputation) revealed similar results. In this sample, the intervention effects on semi-blinded clinician-rated inattentive symptoms, parent-rated DBD-related impairment, and parent-rated negative parenting behaviors were significant (see Table S11), while the effects on semi-blinded clinician-rated overall ADHD symptoms and ADHD-related impairment and on parent-rated functional impairment narrowly missed significance. Given that the respective small to moderate effect sizes were nearly identical to those detected in the ITT sample, the non-significance of the latter effects might be due to power issues (for the sample sizes for the individual analyses, see Table S11).

### Full responders to TASH: changes throughout the course of study

**Fig. 3:**
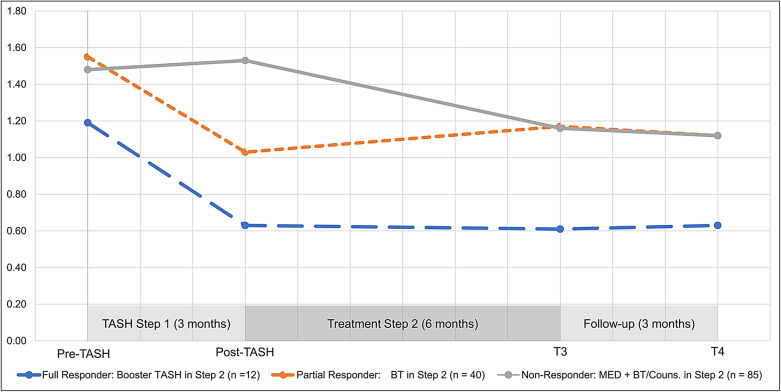
Changes in ADHD symptoms (semi-blinded clinician ratings) throughout the course of study. TASH = telephone-assisted self-help, BT = behavior therapy, MED + BT/Couns. = pharmacotherapy plus behavior therapy/counseling; Pre-TASH = assessment point before TASH intervention (TASH condition: baseline; waitlist control group: after three-month waiting period/before TASH), Post-TASH: assessment point after three-month TASH intervention, T3 = assessment point after six-month treatment in Step 2 (TASH, BT or MED + BT/Couns.), T4 = three-month follow-up assessment point; n = sample size. The error bars represent standard errors

In line with expectation, on an observational level, full responders to TASH demonstrated a large, significant pre-post reduction in ADHD symptom severity (semi-blinded clinician ratings) during Step 1 and a stable ADHD symptom level during Step 2 (TASH booster sessions) and the three-month follow-up period (for detailed results of these within-group analyses, see Fig. 3 and Table S12). The development on most of the other outcomes generally followed this pattern (see Table S12), although the changes during Step 1 did not reach significance for the majority of variables, potentially due to the small sample size of this group. As few teacher ratings were available (*n* < 10), the models could not be analyzed for the teacher-rated outcomes.

The median CGI severity score was 2.00 (*IQR* = 0.00) at the beginning of Step 2, 3.00 (*IQR* = 2.50) at T3, and 2.00 (*IQR* = 2.00) at T4 (seven-point scale, with “2” = borderline mentally ill and “3” = mildly ill). The median CGI improvement score was 2.00 (*IQR* = 0.75) at T3 and 2.00 (*IQR* = 1.00) at T4 (seven-point scale, with “2” = much improved).

### Partial responders to TASH: changes throughout the course of study

The exploratory analyses of the within-group changes in partial responders to TASH throughout the study revealed that these showed a large, significant reduction in ADHD symptom severity (semi-blinded clinician ratings) during Step 1, a slight but non-significant increase in ADHD symptom severity during Step 2 (BT), and a stable symptom level during the follow-up period (see Fig. 3 and Table S13). For the secondary outcomes, we observed mostly small to moderate improvements during Step 1, with significant effects for semi-blinded-clinician-rated ADHD-related impairment and DBD symptoms, for parent-rated ADHD symptoms, ADHD-related impairment, DBD symptoms, functional impairment, and negative parenting behaviors, and for teacher-rated ADHD and DBD symptoms. There were no significant changes in any of the secondary outcomes during Step 2, with the exception of a significant pre-post change in teacher-rated ADHD-related impairment. During the follow-up period, we found small, significant improvements in parent-rated DBD-related impairment, functional impairment and negative parenting behaviors, but no significant changes in any of the other secondary outcomes (see Table S13). Analyses in the PP sample (*n* = 17) yielded similar results (see Table S14). Due to the non-randomized, observational nature of the second study step, the observed changes are susceptible to regression to the mean or selection effects and may not necessarily reflect true change.

The median CGI severity score was 3.00 (*IQR* = 1.00) at the beginning of Step 2, 4.00 (*IQR* = 1.75) at T3, and 3.00 (*IQR* = 1.00) at T4 (“3” = mildly ill, “4” = moderately ill). The median CGI improvement score was 2.50 (*IQR* = 1.00) at T3 and 2.00 (*IQR* = 1.00) at T4 (“2” = much improved, “3” = minimally improved).

### Non-responders to TASH: changes throughout the course of study

The exploratory analyses of within-group changes throughout the course of study in non-responders to TASH, who participated in heterogeneous treatments in Step 2 (medication, BT or counseling, or a combination of both), yielded no significant change in semi-blinded clinician-rated ADHD symptoms during Step 1, but a large, significant reduction in ADHD symptom severity during Step 2 (ITT analysis). During the follow-up period, the symptom level remained stable (see Table S15 and Fig. 3). Regarding the secondary outcomes, there were significant changes on few variables during Step 1. That is, the analyses yielded significant changes in parent-rated inattention symptoms and DBD-related impairment, internalizing symptoms, negative parenting behaviors, and parental self-efficacy, and in teacher-rated ADHD symptoms, ADHD-related impairment and DBD-related impairment (mostly small effects). During Step 2, significant, albeit mostly small to moderate improvements emerged for most of the outcomes. During the follow-up period, we found no significant changes in any of the semi-blinded clinician- or parent-rated outcomes, but significant changes emerged in teacher-rated hyperactive-impulsive symptoms and overall ADHD symptoms (see Table S15). Like all analyses of the Step 2 treatments, given their exploratory character and the lack of a control group, these analyses are vulnerable to regression to the mean and potential selection bias and have to be interpreted cautiously.

The median CGI severity score was 4.00 (*IQR* = 1.00) at the beginning of Step 2 and at T3 and T4. The median CGI improvement score was 2.00 (*IQR* = 1.00) at both T3 and T4.

### Parental satisfaction with the interventions

The parents expressed moderate to high overall satisfaction with all study conditions after Step 1 and Step 2 (mean overall satisfaction score ranging from 3.37 [MED + BT/Couns.] to 3.92 [booster TASH], possible range 1.00 to 5.00; see Table S16). Consistent across all study conditions, parents expressed lower satisfaction with the improvement of their child’s concentration compared to other items. Satisfaction with the improvement of distress in the family environment was comparatively higher. Moreover, a large proportion of families in the TASH and BT conditions expressed rather high satisfaction with how the intervention had helped them to deal with their problems more effectively (see Table S16).

### Psychological side effects

Parents reported rather few psychological side effects for themselves and for their children across all interventions (see Table S17). On a descriptive level, patterns of side effects were quite similar between interventions. Among the most frequently mentioned psychological side effects across all interventions were a lack of leisure time or limited contacts with peers both for the parents and the children, increased conflicts both for the parents and the children, and side effects relating to time and organizational burden for the parents. Moreover, parents reported irritable or bad mood in about one fifth of the children in the BT condition. Importantly, on a descriptive level, more parents reported time and organizational burden for the interventions involving personal contact (BT, MED + BT/Couns.) than for TASH.

## Discussion

The present study examined the efficacy of TASH and changes during subsequent adaptive treatment in a sample of school-age children with mild to moderate symptoms of ADHD. This is one of the first studies to comprehensively examine an adaptive stepped-care approach in school-age children with mild to moderate ADHD, which is recommended in several treatment guidelines [17, 20].

The feasibility of the stepped-care approach is supported by low dropout rates, moderate to high satisfaction ratings, and few reported psychological side effects for the interventions used in this study, although on a descriptive level, treatment adherence in terms of completed sessions and continuation rates appeared to be higher in the first study step compared to the second.

The results from the RCT in Step 1 suggest that TASH is not effective in reducing child ADHD symptoms as rated by a blinded clinician, which was the primary outcome. In line with this finding, previous studies and meta-analyses mostly did not find any significant effects of self-help or face-to-face behavioral interventions on blinded or “probably blinded” outcomes (e.g. teacher ratings, observations of child behavior) [1, 4, 9, 10].

On the other hand, regarding the secondary outcomes, we found significant effects of TASH on semi-blinded clinician ratings of ADHD symptoms and ADHD-related impairment as well as on parent-rated DBD-related impairment and negative parenting behaviors. This is consistent with previous studies and meta-analyses, which have quite consistently reported effects of both self-directed online interventions and bibliotherapy on measures of impairment and parenting behaviors [9, 10, 36]. Several authors have emphasized that the reduction of impairment is at least as important as symptom improvements, given that the majority of behavioral interventions target impairment in specific domains rather than overall symptom-related improvement [2]. Moreover, negative parenting behaviors are supposed to play a role in the development and maintenance of externalizing behavior problems [37] and have been found to mediate the effects of telephone-assisted self-help in previous studies [38]. Therefore, it is conceivable that changes in negative parenting behaviors might lead to changes in ADHD and especially ODD symptoms in the longer term.

Previous studies have additionally reported effects of self-help interventions, including TASH, on parent-rated ADHD and ODD symptoms [9, 10, 39], which were not found in the present study. This discrepancy might be attributable to the relatively short intervention period in this study.

The finding of effects of TASH on semi-blinded (secondary outcome), but not on blinded ratings of ADHD symptoms (primary outcome), might point to the higher power of the analyses of semi-blinded ratings, which were based on a larger sample, but might also hint at bias associated with the semi-blinded ratings. Specifically, while blinded clinicians saw symptomatic improvements in both the TASH group and the WCG, semi-blinded clinicians only perceived such improvement in the TASH condition. Thus, the semi-blinded clinicians rated the WCG in a more hypothesis-consistent manner. Notably, similar to the blinded clinician ratings, no significant effect of TASH on parent-rated ADHD symptoms emerged. The absence of effects in blinded clinician ratings, which are probably less biased than the semi-blinded ones, and in parent ratings suggests that the TASH intervention has no substantial effect on child ADHD symptoms, despite the significant result for the semi-blinded ratings.

On the other hand, the above-mentioned effects on impairment and negative parenting behaviors, in combination with the clinical importance of impairment and the potential role of negative parenting behaviors in mediating treatment effects in the longer term, suggest that TASH could be a useful initial treatment for children with mild-to-moderate ADHD, at least with regard to these variables. This becomes even more striking in light of the often-reported undersupply of local treatment options and the few side effects and low burden associated with TASH reported in the present study, particularly the low time and organizational burden. Especially in cases of long waiting periods or other barriers to treatment access, TASH might be a viable, well-tolerated, low-threshold intervention to start with. The results of the RCT in Step 1 do not seem to be affected by the use of medication or other additional treatments, as this factor was similar in both study conditions.

Some children, albeit a small proportion, fully responded to TASH (8.8%). The vast majority of children, however, showed a partial (29.2%) or non-response (62.0%) to the intervention.

Full responders to TASH showed a stable level of symptoms when receiving a low dose of booster sessions during Step 2, possibly hinting at the utility of these booster sessions. However, this finding is limited by the lack of an untreated control condition, and future studies have to determine whether the stability of symptoms observed in the booster phase is actually attributable to the booster sessions. In partial responders to TASH, further treatment with more intensive multimodal BT did not yield any meaningful additional effects. Although many children in this group participated in fewer sessions than planned, there were also no meaningful pre-post changes in any variables when considering only those children who received a predefined minimum number of sessions (PP sample). However, non-responders to TASH showed significant improvements in several outcome variables, including clinician-rated ADHD symptoms (semi-blinded ratings), during Step 2. It should be noted that as far as we are aware, a substantial percentage of the children in this group did not receive any medication during Step 2, and the non-responders received even fewer sessions of additional BT/counselling than the partial responders. Thus, the larger pre-post improvements in this group compared to the partial responders might be due to the greater scope for improvement. Although the heterogeneous treatment in this group, with large variability in the number of BT/counselling sessions and varying medication use, is close to clinical practice and although the observational findings might therefore have high external validity, this heterogeneity also limits the ability to assess the potential benefits of a specific treatment following TASH. Notably, the large number of missing values regarding the use of medication and the lack of control groups further limit the interpretability of the findings for the Step 2 interventions. It is conceivable that the observed changes may reflect regression to the mean rather than actual effects, and the results might further be biased by selection effects related to group allocation based on prior treatment response. The findings for the Step 2 interventions can therefore only be considered exploratory and tentative, and the use of these interventions following TASH requires further research.

One potential reason for the relatively low number of BT sessions in both partial and non-responders to TASH might be that the families tended to have a rather low level of burden, as the sample consisted of children with mild to moderate ADHD symptoms. Future research could examine whether treatment outcomes might be enhanced by measures to improve treatment engagement and adherence for parents and teachers (e.g., offering video sessions to reduce barriers to participation), by a greater involvement of teachers, or by measures to facilitate the implementation of interventions in daily life (e.g., through smartphone apps). Moreover, the introduction of pharmacotherapy might be an option to enhance treatment outcomes in medication-naïve children with persistent ADHD symptoms and impairment despite BT. In the present study, however, many parents refused medication for their children, and low medication compliance is a general challenge in ADHD treatment [40].

The present findings and conclusions need to be considered in light of several limitations. First, the strict inclusion criteria (such as the exclusion of children with certain comorbidities), the high percentage of children in the present sample with the ADHD inattentive subtype, which is possibly due to the consideration of individuals with mild to moderate symptoms, and the rather high education of many participating parents might limit the generalizability of the findings. Second, the rather low number of completed sessions for BT in the second step, both in partial and non-responders to TASH, and the large amount of missing data regarding the medication status of the non-responders to TASH render it difficult to disentangle treatment-specific effects. Third, the lack of untreated control groups limits the ability to causally attribute changes in the different response groups to the respective interventions in Step 2. Bias is conceivable, e.g. due to regression to the mean. Fourth, the incorrect classification of the response to TASH in eleven children might have influenced the findings for the Step 2 treatments. However, PP analyses for the partial responders to TASH, which excluded these cases, yielded similar results to the ITT analyses.

## Conclusions

For clinical practice and future research, we would like to draw the following, though preliminary conclusions:

- TASH is not effective in reducing blinded clinician-rated child ADHD symptoms (primary outcome). However, it is associated with at least partial response in a substantial proportion of children (based on semi-blinded clinician ratings) and shows effects on impairment and parenting practices. Although probably not effective in reducing child ADHD symptoms, TASH may be a useful initial treatment in school-age children with mild to moderate symptoms of ADHD in terms of these specific variables. In view of the larger effects found in previous studies with longer treatment duration [36], an extension of the three-month intervention period could be considered, at least for children with a partial treatment response. Potential benefits of TASH with longer treatment duration as initial treatment in a stepped-care approach require further examination in future studies.
- Regular booster sessions in participants with a full response to TASH might help to maintain the treatment effects.
- In the present study, children with a partial response to TASH did not demonstrate pre-post improvements following further treatment with multimodal behavioral face-to-face therapy, thereby generating the hypothesis for future trials that this treatment may not be indicated for this particular patient group (though it should be noted that many families in the present study received only a limited number of sessions). Given the observational and uncontrolled nature of the second study step, as well as the heterogeneity in the number of sessions received, these findings are not intended to provide confirmatory evidence but should be considered preliminary at this stage of research. Moreover, future research should examine whether treatment adaptations (e.g., measures to enhance motivation) or the (alternative or additional) use of pharmacotherapy may enhance treatment adherence and outcomes.
- Considering non-responders to TASH, the findings of the present study give rise to the hypothesis for future research that intensified treatment, including multimodal behavioral face-to-face interventions, medication or a combination of both may be a useful treatment for this particular group. In the present study, symptomatic pre-post improvements were observed under heterogeneous treatment conditions, with some of the children participating in behavioral treatment, some receiving medication, and others receiving combined treatment. Under these circumstances, and in light of the observational nature of Step 2, it is difficult to draw firm conclusions regarding subsequent treatment in non-responders to TASH; further studies are required to determine which specific treatment is most appropriate for this patient group.

The findings for all Step 2 treatments are limited by the lack of an untreated control condition and are susceptible to regression to the mean or selection effects related to classification into response groups based on prior treatment response; the results for the interventions in partial responders and non-responders to TASH are further limited by the variability in the number of sessions and the use of medication. Therefore, the findings can only be regarded tentative and hypothesis-generating; future studies have to examine whether observed changes (or stability) are actually attributable to the interventions.

In summary, the present study found that the 3-month TASH intervention did not improve blinded clinician-rated ADHD symptoms (primary outcome) compared to a WCG in school-age children with mild to moderate ADHD. However, exploratory analyses indicated effects on impairment and negative parenting behaviors. The question of optimal treatment following TASH remains uncertain, particularly in light of the aforementioned limitations related to the study design and the heterogeneity of the interventions actually received.

## Supplementary Information

Supplementary Material 1.

## Data Availability

Pseudonymized patient data will be made available upon request to achieve aims in an approved proposal. Proposals can be submitted by the sponsors, all participating study centers, and by any entity commissioned by the sponsors for the purpose of scientific evaluation. Proposals should be directed to anna.kaiser@zi-mannheim.de; to gain access, the proposals have to be approved by the publication board(s) of the respective trial(s) and data requestors will need to sign a data access agreement. The study protocol of the study including the statistical analysis plan is available at: 10.1186/s12888-017-1433-9.

## Abbreviations

ADHD: Attention-deficit/hyperactivity disorder
ANCOVA: Univariate analysis of covariance
BT: Behavior therapy
CGI: Clinical Global Impression Scale
DBD: Disruptive behavior disorders
DCL-ADHD: Diagnostic Checklist for ADHD
FAS: Full analysis sample
ITT: Intention-to-treat
MED + BT/Couns.: Medication plus behavior therapy or plus counseling
PP: Per-protocol
TASH: Telephone-assisted self-help
RCT: Randomized controlled trial
WCG: Waitlist control group

## Declarations

### Accordance with CONSORT guidelines

The reporting of this study followed the CONSORT Guidelines (see Table S18).
