# Supplementary material for: Efficacy of a stepped-care approach for school-age children with mild to moderate ADHD (ESCAschool-moderate): an adaptive intervention study including a randomized controlled trial
Source: Child Adolesc Psychiatry Ment Health. 2026 Jul 23;20:102. doi: 10.1186/s13034-026-01115-3 (PMC13452123; doi:10.1186/s13034-026-01115-3)
Supplement: Supplementary file 1 [file 13034_2026_1115_MOESM1_ESM.pdf]

Online supplement for the article:

Efficacy of a Stepped Care Approach for School-Age Children with Mild to Moderate ADHD (ESCAschool-moderate): An Adaptive Intervention Study Including a Randomized Controlled Trial

Contents

Table S1. Deviations from the study protocol and major protocol violations 2

Table S2. Telephone-assisted self-help (TASH): Contents of the parent- and the teacher-directed booklets 3

Table S3. Behavior Therapy: Parent-, child-, and teacher-focused components of the behavior therapy intervention 4

Table S4. Counseling: Parent-, child-, and teacher-focused components of the counseling sessions 5

Table S5. Description and Psychometric Properties of the Outcome Measures 6

Table S6. Sociodemographic Characteristics at T1 9

Table S7. Clinical Characteristics at T1 and T2 10

Table S8. Medication 11

Table S9. Utilization of additional treatments 12

Table S10. Results of ANCOVAs for the group comparisons regarding the mean change from baseline (T1) to 3 months (T2): intention-to-treat sample; missing values imputed by Bayesian Stochastic regression 13

Table S11. Results of ANCOVAs for the group comparisons regarding the mean change from baseline (T1) to 3 months (T2): per-protocol analysis 15

Table S12. Results of analyses of piecewise mixed-effects models for repeated measures for the changes within the full responders to TASH from T1 to T4: intention-to-treat sample 17

Table S13. Results of the analyses of piecewise mixed-effects models for repeated measures for the changes within the partial responders to TASH from T1 to T4: intention-to-treat sample 19

Table S14. Results of the analyses of piecewise mixed-effects models for repeated measures for the changes within the partial responders to TASH from T1 to T4: per-protocol sample 21

Table S15. Results of the analyses of piecewise mixed-effects models for repeated measures for the changes within the non-responders to TASH from T1 to T4: intention-to-treat sample 23

Table S16. Parental satisfaction with the interventions 25

Table S17. Psychological side effects 26

Table S18. CONSORT 2025 checklist of information to include when reporting a randomised trial 27

References 30

Table S1. Deviations from the study protocol and major protocol violations

**Deviations from the published study protocol**	
Domain	
Interventions	Originally, non-responders to TASH were supposed to receive pharmacotherapy plus behavior therapy in the second study step. Due to an unexpectedly high number of non-responders and lack of resources at some of the participating centers, this was changed to the following formulation: “Non-responders with persisting moderate ADHD after Step 1 will receive pharmacotherapy (if indicated according to clinical guidelines) and additionally behavior therapy or counseling according to center standards (if indicated).”
**Major protocol violations**	
Domain	
Intervention, Step 1	10 children in the TASH condition received less than 7 parent-directed telephone counseling sessions.
Intervention, Step 1	20 children randomized to the waitlist control condition received less than 7 parent-directed telephone counseling sessions (TASH after waiting period).
Classification of response to TASH	5 children were falsely classified as non-responders to TASH, but were actually partial responders.<br>6 children were falsely classified as partial responders to TASH, but were actually non-responders.
Interventions, Step 2	16 children in the behavior therapy condition (partial responders to TASH) received less than 14 sessions of behavior therapy.

Note. TASH = telephone-assisted self-help

Table S2. Telephone-assisted self-help (TASH): Contents of the parent- and the teacher-directed booklets

Booklet no.	Content
**Parent program**	
1	- Definition of individual target problems for the intervention<br>- Psychoeducation about coercive parent-child interactions
2	- Psychoeducation about ADHD and related problems (symptoms, comorbidities, developmental course, treatment options)
3	- Encouragement of positive parent-child interactions
4	- Reconsideration and implementation of family rules
5	- Effective methods of communicating demands, praise, and appropriate positive consequences of adhering to rules
6	- Appropriate negative consequences of breaking rules
7	- Implementation of reward systems (token economies or response cost)
8	- Development of daily structures, leisure activities of the child (e.g., use of media), and stress reduction techniques for the parents
**Teacher program**	
Booklet no.	Content
1	- Psychoeducation about ADHD and related problems (symptoms, comorbidities, developmental course, treatment options)<br>- Definition of individual target problems for the intervention<br>- Psychoeducation about coercive teacher-child interactions
2	- Establishing a supportive environment at school<br>- Encouragement of positive teacher-child interactions
3	- Definition of classroom rules<br>- Definition of individual rules<br>- Effective methods of communicating demands
4	- Praise and appropriate positive consequences of adhering to rules<br>- Appropriate negative consequences of breaking rules<br>- Implementation of reward systems (token economies or response cost)

Notes. Besides the booklets, the TASH parent program comprised ten accompanying, approximately weekly telephone consultations of about 20 to 30 minutes. The TASH teacher program included four additional telephone consultations, each lasting up to 60 minutes. The telephone consultations served to support the parents (or teachers, respectively) in implementing the techniques described in the booklets. The parent booklets were revised versions of booklets that had demonstrated efficacy and effectiveness in previous trials (Döpfner et al., 2021; Dose et al., 2017; Ise et al., 2015; Kierfeld et al., 2013; Mokros et al., 2015); the teacher program was newly developed for the present study.

Table S3. Behavior Therapy: Parent-, child-, and teacher-focused components of the behavior therapy intervention

**Introductory sessions (sessions 1–5)**		
Parent	Child	Teacher
• Exploration of current externalizing symptoms of the child<br>• Exploration of specific current ADHD symptoms of the child (mandatory; video-recorded for supervision purposes)<br>• Psychoeducation on ADHD, comorbidities, prevalence rates, etiology, developmental course, treatment options	• Exploration of and psychoeducation on ADHD<br>• Clinical observation of homework situation (mandatory; video-recorded for supervision purposes)<br>• Resource-activating interventions, interventions to promote self-esteem	• Exploration of current externalizing symptoms of the child at school<br>• Psychoeducation on ADHD, comorbidities, prevalence rates, etiology, developmental course, treatment options
**Basic sessions (sessions 6–10)**		
Parent	Child	Teacher
• Focusing on positive experiences with the child<br>• Implementing clear rules and boundaries<br>• Making effective requests<br>• Social reinforcement<br>• Appropriate negative consequences of undesirable behaviors	• Organizational skills training: self-observation<br>• Organizational skills training: self-regulation strategies<br>• Organizational skills training: self-instruction	• Promoting positive parent-child interactions<br>• Establishing a favorable school environment
**In-depth sessions (sessions 11–20)**		
Parent (and child)	Teacher	
• Development of positive play interactions<br>• Development and implementation of token economies<br>• Response cost<br>• Time-out<br>• Parental self-care<br>• Promoting the child's strengths and interests<br>• Managing behavioral problems in the homework situation<br>• Managing behavioral problems in public<br>• In-depth module for improving organizational skills in selected problem areas<br>• Counseling on non-externalizing/emotional problems of the child	• Implementing clear rules and boundaries for the individual child and class<br>• Making effective requests<br>• Positive and negative consequences<br>• Token economies and response cost	

Notes. The interventions were primarily based on the German "Therapy Program for Children with Hyperactive and Oppositional Problem Behaviors" (THOP; Döpfner et al., 2019), which has been reported to be effective (Döpfner et al., 2004, 2015, 2021). In addition, child-focused interventions for training organizational and planning skills were taken from the German "Therapy Program to Enhance Organizational Skills, Concentration and Impulse Control" (THOKI-ADHD; Braun & Döpfner, 2024). Previous studies have likewise demonstrated the effectiveness of the treatment strategies used in this manual (Abikoff et al., 2013; Langberg et al., 2012).

One component could be applied in several sessions and more than one component could be applied in one session. Therapists were advised to discuss the contents of the introductory sessions and of the basic sessions with all families, if possible. The contents of the in-depth sessions were chosen depending on the families' individual needs. The choice of treatment components and the ratio of child sessions to parent/teacher sessions (approximately 1:2) were determined with the support of a senior supervisor and in consideration of the child's age and the areas in which problem behaviors occurred (home, school, during sessions).

Table S4. Counseling: Parent-, child-, and teacher-focused components of the counseling sessions

Introductory sessions		
Parent	Child	Teacher
• Exploration of current symptoms of the child	• Exploration of and psychoeducation on ADHD<br>• Resource-activating interventions, interventions to promote self-esteem<br>• Counseling on current externalizing behavior problems	• Exploration of current symptoms of the child at school

In-depth sessions	
Parent (and child)	Teacher
• Focusing on positive experiences with the child<br>• Implementing clear rules and boundaries<br>• Making effective requests<br>• Social reinforcement<br>• Appropriate negative consequences of undesirable behaviors<br>• Development of positive play interactions<br>• Development and implementation of token economies<br>• Response cost<br>• Parental self-care<br>• Promoting the child's strengths and interests<br>• Counseling on non-externalizing/emotional problems of the child	• Promoting positive parent-child interactions<br>• Establishing a favorable school environment<br>• Implementing clear rules and boundaries for the individual child and class<br>• Making effective requests<br>• Positive and negative consequences<br>• Token economies and response cost

Note. One component could be applied in several sessions and more than one component could be applied in one session. Therapists were advised to discuss the contents of the introductory sessions and of the basic sessions with all families, if possible. The contents of the in-depth sessions were chosen depending on the families' individual needs.

Table S5. Description and Psychometric Properties of the Outcome Measures

Outcome	Rater	Measure	Description	Psychometric properties
ADHD symptoms	Blinded and semi-blinded clinician	Diagnostic Checklist for Attention-Deficit/Hyperactivity Disorder (DCL-ADHD; Döpfner & Görtz-Dorten, 2017)	• Diagnostic checklist to assess symptoms of ADHD according to DSM-5 and ICD-10<br>• 18 items to be rated on a 4-point Likert-type scale ranging from 0 (*not at all*) to 3 (*very much*), which can be summarized in a total score<br>• Two symptom-related subscales: Inattention, Hyperactivity/Impulsivity<br>• 5 additional items to assess ADHD-related impairment	• Internal consistency: Cronbach's α = 0.84 for the total score; α = 0.71 for the Inattention subscale and α = 0.87 for the Hyperactivity/Impulsivity subscale (Thöne et al., 2020)<br>• Good to excellent interrater reliability (intraclass coefficient ≥ 0.83) subscale (Thöne et al., 2020)<br>• Convergent and divergent validity subscale (Thöne et al., 2020)<br>• Internal consistencies for the blinded ratings in the present sample: DCL-ADHD total score α = 0.74, Inattention α = 0.67, Hyperactivity/Impulsivity α = 0.81, Impairment α = 0.51<br>• Internal consistencies for the semi-blinded ratings in the ESCAschool total sample (mild-to-moderate + severe): DCL-ADHD total score α = .81, Inattention α = 0.67, Hyperactivity/Impulsivity α = 0.86, Impairment α = 0.59
ADHD symptoms	Parent, teacher	Symptom Checklist for Attention-Deficit/Hyperactivity Disorder (SCL-ADHD; Döpfner & Görtz-Dorten, 2017)	• Questionnaire to assess symptoms of ADHD according to DSM-5 and ICD-10<br>• 18 symptom-related items to be rated on a 4-point Likert-type scale ranging from 0 (*not at all*) to 3 (*very much*), which can be summarized in a total score<br>• Two symptom-related subscales: Inattention, Hyperactivity/Impulsivity<br>• 5 additional items to assess ADHD-related impairment	• Factorial validity (Döpfner & Görtz-Dorten, 2017; Erhart et al., 2008)<br>• Internal consistency: Cronbach's α > 0.80 for all subscales and the total score[1] (Döpfner & Görtz-Dorten, 2017; Erhart et al., 2008)<br>• Internal consistencies for the parent ratings in the present sample: SCL-ADHD total score α = 0.89, Inattention α = 0.84, Hyperactivity/Impulsivity α = 0.90, Impairment α = 0.78<br>• Internal consistencies for the teacher ratings in the present sample: SCL-ADHD total score α = 0.90, Inattention α = 0.87, Hyperactivity/Impulsivity α = 0.92, Impairment α = 0.77
Symptoms of disruptive behavior disorders	Blinded and semi-blinded clinician	Diagnostic Checklist for Disruptive Behavior Disorders (DCL-DBD; Döpfner & Görtz-Dorten, 2017)	• Diagnostic checklist to assess symptoms of oppositional defiant disorder (ODD), disruptive mood dysregulation disorder (DMDD), and conduct disorder (CD) according to ICD-10 and DSM-V as well as characteristics of limited prosocial emotions<br>• 26 symptom-related items to be rated on a 4-point Likert-type scale ranging from 0 (*not at all*) to 3 (*very much*)<br>• 5 additional items to assess impairment related to symptoms of disruptive behavior disorders<br>• Four symptom-related subscales: ODD, DMDD, CD, limited prosocial emotions<br>• For the present study, we considered the total score as well as the ODD (8 items) and Impairment subscales.	• Internal consistency: Cronbach's α = 0.60–0.86[2]<br>• Very good to excellent interrater reliability (intraclass coefficient ≥ 0.90; Thöne et al., 2020)<br>• Convergent and divergent validity (Töne et al., 2020)<br>• Internal consistencies for the blinded ratings in the present sample: DCL-DBD total score α = 0.86, ODD α = 0.80, Impairment α = 0.67<br>• Internal consistencies for the semi-blinded ratings in the present sample: DCL-DBD total score α = 0.86, ODD α = 0.78, Impairment α = 0.82
Symptoms of disruptive behavior disorders	Parent, teacher	Symptom Checklist for Disruptive Behavior Disorders (SCL-DBD; Döpfner & Görtz-Dorten, 2017)	• Questionnaire to assess symptoms of oppositional defiant disorder (ODD), disruptive mood dysregulation disorder (DMDD), and conduct disorder (CD) according to ICD-10 and DSM-V as well as characteristics of limited prosocial emotions	• Internal consistency: Cronbach's α > 0.80 for all subscales and the total score (Görtz-Dorten & Döpfner, 2017; Görtz-Dorten et al., 2014)

Table S5. Description and Psychometric Properties of the Outcome Measures

Outcome	Rater	Measure	Description	Psychometric properties
			• 28 symptom-related items to be rated on a 4-point Likert-type scale ranging from 0 (*not at all*) to 3 (*very much*)<br>• 5 additional items to assess impairment related to symptoms of disruptive behavior disorders<br>• Four symptom-related subscales: ODD, DMDD, CD, limited prosocial emotions<br>• For the present study, we considered the total score as well as the ODD (8 items) and Impairment subscales.	• Factorial, convergent; and divergent validity (Görtz-Dorten & Döpfner, 2017; Görtz-Dorten et al., 2014)<br>• Internal consistencies for the parent ratings in the present sample: SCL-DBD total score α = 0.93, ODD α = 0.90, Impairment α = 0.87<br>• Internal consistencies for the teacher ratings in the present sample: SCL-DBD total score α = 0.94, ODD α = 0.91, Impairment α = 0.89
Clinical global impression	Blinded and semi-blinded clinician	Clinical Global Impression Scale (CGI; Guy, 1976)	• Two 1-item scales: severity scale (CGI-S) and improvement scale (CGI-I) to globally assess a patient's severity of illness at the time of assessment and the improvement of the illness relative to a baseline assessment<br>• Items to be rated on a 7-point scale ranging from 1 (*normal, not at all ill / very much improved*) to 7 (*among the most extremely ill patients / very much worse*)	• Commonly used measure in clinical trials<br>• Several studies have yielded support for the measure's validity
Broad spectrum of child behavioral and emotional problems	Parent	Revised German version of the Child Behavior Checklist for Ages 6–18 (CBCL 6–18R; Achenbach & Rescorla, 2001; Döpfner et al., 2014)	• Questionnaire to assess behavioral and emotional problems<br>• 120 items to be rated on a 3-point scale from 0 (*not true*) to 2 (*very true or often true*)<br>• Syndrome scales: Aggressive behavior, withdrawn/depressed, somatic complaints, anxious/depressed, social problems, attention problems, rule-breaking behavior<br>• Three superordinate scales: internalizing problems, externalizing problems, total problems<br>• For the present study, we only considered the superordinate scale on internalizing problems.	• Factorial validity of subscales<br>• Internal consistency: CBCL total score: Cronbach's α = 0.93 [19]<br>• Internal consistency in the present sample: α = 0.80
Functional impairment	Parent	Modified German version of the Weiss Functional Impairment Rating Scale - Parent Report (WFIRS-P-M; Dose et al., 2019)	• Questionnaire to assess ADHD-related functional impairment<br>• 40 items to be rated on a 4-point Likert-type scale ranging from 0 (*never or not at all*) to 3 (*very often or very much*)<br>• Five subscales: Family, Learning and School, Self-Concept, Life Skills, Social Activities<br>• For the present study, we only considered the total score.	• Original version (WFIRS-P): factorial validity, internal consistency, and test-retest reliability (Weiss et al., 2018)<br>• Modified German version: factorial and divergent validity, internal consistency: Cronbach's α = 0.73–0.87 for the subscales and α = 0.92 for the total score (Dose et al., 2019)<br>• Internal consistency in the present sample: α = 0.93
Quality of life	Parent	German questionnaire for measuring health-related quality of life in children and adolescents (KIDSCREEN-10; KIDSCREEN Group Europe, 2006; Ravens-Sieberer et al., 2010)	• Questionnaire to assess health-related quality of life<br>• 10 items to be rated on a 5-point Likert-type scale ranging from 1 (*never/not at all*) to 5 (*very much/always*)<br>• Items can be summarized in a total score	• Internal consistency (Ravens-Sieberer et al., 2010)<br>• Test-retest reliability (Ravens-Sieberer et al., 2010)<br>• Discriminant validity (Ravens-Sieberer et al., 2010)<br>• Internal consistency in the present sample: α = 0.80
Parental self-efficacy	Parent	German version of the *Problem Setting and Behavior Checklist* (PSBC; Naumann et al., 2010; Sanders et al., 2000)	• 27 items describing typical problem situations<br>• Items to be rated on a 4-point Likert-type scale ranging from 1 (*I cannot cope with the situation*) to 4 (*I can cope with the situation very well*)<br>• Items can be summarized in a total score	• Internal consistency (Naumann et al., 2010)<br>• Internal consistency in the present sample: α = 0.93

Table S5. Description and Psychometric Properties of the Outcome Measures

Outcome	Rater	Measure	Description	Psychometric properties
Positive and negative parenting practices	Parent	Assessment Scale of Positive and Negative-Inept Parenting Behavior (FPNE; Holas et al., 2024)	• Questionnaire to assess parenting practices<br>• 23 items to be rated on a 4-point Likert-type scale ranging from 0 (*never*) to 3 (*very often*)<br>• Two subscales: positive parenting (13 items), negative parenting (10 items)	• Factorial validity (Holas et al., 2024)<br>• Internal consistency) (Holas et al., 2024; Naumann et al., 2010)<br>• Convergent validity (Holas et al., 2024)<br>• Internal consistency of the positive parenting scale in the present sample: $\alpha = 0.84$<br>• Internal consistency of the negative parenting scale in the present sample: $\alpha = 0.69$

Table S6. Sociodemographic Characteristics at T1

	TASH (*n* = 80)		WCG (*n* = 83)	
	n	%	n	%
Child gender: male	61	76.3	66	79.5
Primary diagnosis n (%)				
– ADHD – combined type	11	13.8	15	18.1
– ADHD – predominantly inattentive type	59	73.8	52	62.7
– ADHD – predominantly hyperactive–impulsive type	10	12.5	16	19.3
Comorbidities n (%)				
Externalizing disorders:				
– Oppositional defiant disorder[a,b]	18	22.8	20	24.4
– Conduct disorder[a,b]	2	2.5	5	6.1
– Disruptive mood dysregulation disorder	2	2.5	7	8.5
Internalizing disorders:				
– Anxiety	2	2.5	6	7.2
– Depression	1	1.3	1	1.2
Other disorders:				
– Tic disorder	5	6.3	4	4.8
– Obsessive-compulsive disorder	0	0.0	1	1.2
Parental education (based on Organization for Economic Co-operation and Development [OECD, 1999)				
– Pre-primary and primary education	0	0.0	0	0.0
– Lower secondary education	4	5.0	3	3.6
– Upper and post-secondary education	39	48.8	47	56.6
– Tertiary education	34	42.5	29	34.9
– Others (foreign qualification)	3	3.8	4	4.8
Receiving unemployment benefit[a]	4	5.0	12	14.5
Migration status[a,b]				
None, native	71	89.9	75	91.5
Second-generation	1	1.3	1	1.2
First-generation	7	8.9	6	7.3
Single parenthood	10	12.5	11	13.3

Note. TASH = telephone-assisted self-help, WCG = waitlist control group, n = sample size, χ^2 = empirical χ^2 value, M = mean, SD = standard deviation, t = empirical t-value, df = degrees of freedom, p = significance value.
[a] one missing value in the TASH group.
[b] one missing value in the WCG.

Table S7. Clinical Characteristics at T1 and T2

	All available data at T1						Data included in the analysis of the RCT in Step 1 (ITT)											
							T1						T2					
	TASH			WCG			TASH			WCG			TASH			WCG		
	n	M	SD	n	M	SD	n	M	SD	n	M	SD	n	M	SD	n	M	SD
Child age	80	8.92	1.43	83	8.80	1.43												
**Blinded clinician**																		
DCL-ADHD Total	74	1.38	0.38	73	1.39	0.39	59	1.37	0.40	58	1.42	0.40	59	1.25	0.43	58	1.29	0.43
DCL-ADHD Impairment	70	1.09	0.48	67	1.12	0.49	55	1.08	0.49	49	1.11	0.40	55	1.00	0.48	49	1.09	0.40
DCL-DBD Total	63	0.45	0.34	63	0.53	0.30	56	0.45	0.34	49	0.54	0.29	56	0.37	0.30	49	0.49	0.32
DCL-DBD ODD	63	0.80	0.55	63	0.90	0.48	56	0.82	0.55	49	0.89	0.48	56	0.66	0.50	49	0.78	0.50
DCL-DBD Impairment	39	0.71	0.51	44	0.79	0.55	51	0.76	0.51	27	0.76	0.54	51	0.68	0.66	27	0.74	0.46
**Semi-blinded clinician**																		
DCL-ADHD Total	80	1.44	0.33	83	1.49	0.31	74	1.44	0.33	74	1.30	0.44	72	1.51	0.31	72	1.48	0.48
DCL-ADHD Impairment	79	1.24	0.38	83	1.28	0.50	73	1.24	0.35	73	1.06	0.51	72	1.28	0.50	72	1.25	0.49
DCL-DBD Total	80	0.49	0.35	82	0.58	0.32	74	0.46	0.33	74	0.38	0.31	70	0.57	0.33	70	0.49	0.32
DCL-DBD ODD	80	0.82	0.54	82	0.95	0.52	74	0.78	0.51	74	0.66	0.46	70	0.92	0.52	70	0.81	0.55
DCL-DBD Impairment	80	0.61	0.63	82	0.65	0.61	73	0.58	0.61	73	0.53	0.57	71	0.66	0.62	71	0.72	0.59
**Parent**																		
SCL-ADHD Total	78	1.47	0.48	83	1.58	0.53	68	1.46	0.48	68	1.38	0.45	68	1.63	0.50	68	1.56	0.51
SCL-ADHD Impairment	74	1.34	0.66	78	1.52	0.66	62	1.32	0.66	62	1.29	0.53	62	1.52	0.65	62	1.50	0.64
SCL-DBD Total	77	0.60	0.43	82	0.71	0.48	68	0.59	0.44	68	0.54	0.40	69	0.70	0.47	69	0.66	0.44
SCL-DBD ODD	77	0.99	0.64	82	1.15	0.72	68	0.99	0.64	68	0.94	0.65	69	1.11	0.71	69	1.10	0.66
SCL-DBD Impairment	74	1.00	0.79	76	1.09	0.71	63	0.98	0.77	63	0.87	0.70	60	1.09	0.73	60	1.23	0.73
CBCL Internalizing	77	8.10	6.57	83	7.33	5.50	68	7.98	6.69	68	8.09	6.95	70	7.54	5.52	70	8.46	6.33
WFIRS Functional Impairment	78	0.77	0.42	83	0.85	0.47	67	0.75	0.40	67	0.70	0.37	69	0.88	0.47	69	0.89	0.45
KIDSCREEN Quality of Life	78	37.93	5.01	78	38.01	4.78	66	37.96	5.00	66	37.87	4.23	65	37.95	4.41	65	37.71	4.36
FPNE Positive Parenting	78	1.89	0.34	83	1.88	0.34	68	1.89	0.39	68	1.94	0.36	70	1.87	0.34	70	1.86	0.29
FPNE Negative Parenting	77	1.92	0.36	82	1.93	0.35	67	1.87	0.34	67	1.75	0.33	69	1.93	0.35	69	1.94	0.37
PSBC Parental Self-efficacy	76	3.14	0.43	81	3.11	0.50	66	3.16	0.42	66	3.27	0.40	68	3.07	0.49	68	3.14	0.46
**Teacher**																		
SCL-ADHD Total	51	1.48	0.60	47	1.48	0.53	33	1.52	0.65	33	1.33	0.65	37	1.40	0.52	37	1.37	0.60
SCL-ADHD Impairment	44	1.72	0.68	41	1.75	0.71	27	1.75	0.79	27	1.57	0.73	29	1.82	0.65	29	1.80	0.61
SCL-DBD Total	52	0.73	0.69	48	0.53	0.45	35	0.80	0.75	35	0.62	0.62	37	0.53	0.44	37	0.54	0.51
SCL-DBD ODD	52	0.89	0.86	48	0.70	0.57	35	0.99	0.94	35	0.80	0.81	37	0.69	0.58	37	0.76	0.71
SCL-DBD Impairment	43	1.47	0.94	40	1.21	0.89	23	1.54	1.05	23	1.24	0.92	28	1.35	0.88	28	1.30	0.82

Note. RCT = randomized controlled trial; ITT = intention-to-treat sample; TASH = telephone-assisted self-help, WCG = waitlist control group; DCL-ADHD = Diagnostic Checklist for Attention-Deficit/Hyperactivity Disorder, DCL-DBD = Diagnostic Checklist for Disruptive Behavior Disorders, ODD = oppositional defiant disorder, SCL-ADHD = Symptom Checklist for Attention-Deficit/Hyperactivity Disorder, SCL-DBD = Symptom Checklist for Disruptive Behavior Disorders, CBCL = Child Behavior Checklist, WFIRS = Weiss Functional Impairment Rating Scale, KIDSCREEN = German questionnaire for the assessment of health-related quality of life, FPNE = German questionnaire for the assessment of positive and negative parenting behaviors, PSBC = German version of the Problem Setting and Behavior Checklist.
n = sample size. M = mean, SD = standard deviation.

Table S8. Medication

	Step 1								
	Total		TASH		WCG		TASH vs. WCG		
	n (total n)	%	n (total n)	%	n (total n)	%	χ^2	df	p
Medication at T1*	8 (163)	4.9	2 (80)	2.5	6 (83)	7.2	1.95	1	0.28
Methylphenidate	7 (163)	3.6	1 (80)	1.3	5 (83)	6.0	NA	NA	NA
Lisdexamfetamine	1 (163)	0.6	1 (80)	1.3	0 (83)	0.0	NA	NA	NA
Medication at T2*	13 (86)	15.1	5 (44)	11.4	8 (42)	19.0	0.99	1	0.38
Methylphenidate	12 (86)	14.0	4 (44)	9.1	8 (42)	19.0	NA	NA	NA
Lisdexamfetamine	1 (86)	1.2	1 (44)	2.3	0 (42)	0.0	NA	NA	NA

	Step 2							
	Total		Booster TASH		BT		MED+ BT/Couns.	
	n (total n)	%	n (total n)	%	n (total n)	%	n (total n)	%
Medication at T1	7 (137)	5.1	0 (12)	0.0	1 (40)	2.5	6 (85)	7.1
Methylphenidate	6 (137)	4.4	0 (12)	0.0	1 (40)	2.5	5 (85)	5.9
Lisdexamfetamine	1 (137)	0.7	0 (12)	0.0	0 (40)	0.0	1 (85)	1.2
Medication at "Step2 Baseline"	12 (74)	16.2	2 (8)	25.0	4 (16)	25.0	6 (50)	12.0
Methylphenidate	11 (74)	14.9	2 (8)	25.0	4 (16)	25.0	5 (50)	10.0
Lisdexamfetamine	1 (74)	1.4	0 (8)	0.0	0 (16)	0.0	1 (50)	2.0
Medication at T3	39 (71)	54.9	2 (8)	25.0	5 (15)	33.3	32 (48)	66.7
Methylphenidate	33 (71)	46.5	1 (8)	12.5	5 (15)	33.3	28 (48)	58.3
Lisdexamfetamine	2 (71)	2.8	1 (8)	12.5	0 (15)	0.0	1 (48)	2.1
Atomoxetine	2 (71)	2.8	0 (8)	0.0	0 (15)	0.0	2 (48)	4.2
Guanfacine	1 (71)	1.4	0 (8)	0.0	0 (15)	0.0	1 (48)	2.1
Medication at T4	40 (67)	59.7	2 (8)	25.0	5 (15)	33.3	33 (44)	75.0
Methylphenidate	36 (67)	53.7	1 (8)	12.5	5 (15)	33.3	30 (44)	68.2
Lisdexamfetamine	2 (67)	3.0	1 (8)	12.5	0 (15)	0.0	1 (44)	2.3
Atomoxetine	2 (67)	3.0	0 (8)	0.0	0 (15)	0.0	2 (44)	4.5

Note. TASH = telephone-assisted self-help, WCG = waitlist control group, T1 = baseline assessment point, T2 = three-month assessment point, Step 2 Baseline = assessment after completion of TASH, i.e. T2 for children in the TASH group during Step 1 and T2b for children in the waitlist control group during Step, T3 = post-assessment after Step 2, T4 = three-month follow-up assessment point, n = number of cases, χ^2 = empirical χ^2 value, df = degrees of freedom, p = significance value.

*There was no significant group difference in methylphenidate dosage at T1 (M_{TASH} = 0.50, SD_{TASH} = 4.47, M_{WCG} = 1.69, SD_{WCG} = 6.95; t = -1.29, df = 161, p = 0.20) or at T2 (MT_{ASH} = 2.73, SD_{TASH} = 10.14, M_{WCG} = 4.64, SD_{WCG} = 10.38; t = -0.87, df = 84, p = 0.39).

Table S9. Utilization of additional treatments

	Step 1								
	Total		TASH (n = 61)		WCG (n = 60)		TASH vs. WCG		
	n	%	n	%	n	%	χ^2	*df*	*p*
Psychotherapy*	1	0.8	0	0.0	1	1.7	1.01	1	> 0.99
(Other) psychiatric, psychological, or pedagogical counseling**	5	4.2	3	5.0	2	3.3	0.21	1	> 0.99
Parent training	2	1.7	1	1.6	1	1.7	0.00	1	> 0.99
Learning therapy/treatment for dyslexia or dyscalculia/attention training	6	5.0	4	6.6	2	3.3	0.67	1	0.68
Neurofeedback	1	0.8	0	0.0	1	1.7	1.03	1	0.50
Ergotherapy	32	26.4	17	27.9	15	25.0	0.13	1	0.84
Speech therapy	8	6.6	3	4.9	5	8.3	0.57	1	0.49
Specific measures/programs at school	7	5.8	5	8.2	2	3.3	1.31	1	0.44
Youth welfare office	3	2.5	2	3.3	1	1.7	0.33	1	> 0.99
Others	4	3.3	2	3.3	2	3.3	0.00	1	> 0.99
At least one additional treatment*	58	49.6	31	53.4	27	45.8	0.69	1	0.46

	Step 2							
	Total		Booster TASH (n = 10)		BT (n = 29)		MED+ BT/Couns (n = 68)	
	n	%	n	%	n	%	n	%
Psychotherapy	6	5.6	1	10.0	3	10.3	2	2.9
(Other) psychiatric, psychological, or pedagogical counseling	6	5.7	1	10.0	1	3.4	4	6.0
Parent training	2	1.9	0	0.0	0	0.0	2	2.9
Learning therapy/treatment for dyslexia or dyscalculia/attention training	8	7.5	0	0.0	4	13.8	4	5.9
Neurofeedback	3	2.8	0	0.0	1	3.4	2	2.9
Ergotherapy	25	23.4	3	30.0	6	20.7	16	23.5
Speech therapy	8	7.5	1	10.0	3	10.3	4	5.9
Specific measures or programs at school	10	9.3	1	10.0	2	6.9	7	10.3
Youth welfare office	1	0.9	0	0.0	0	0.0	1	1.5
Others	6	5.6	0	0.0	1	3.4	5	7.4
At least one additional treatment	59	55.7	7	70.0	15	51.7	37	55.2

	Follow-Up							
	Total		Booster TASH (n = 10)		BT (n = 25)		MED+ BT/Couns (n = 62)	
	n	%	*n*	%	*n*	%	*n*	%
Psychotherapy	28	28.6	1	10.0	10	38.5	17	27.4
(Other) psychiatric, psychological, or pedagogical counseling***	5	5.2	0	0.0	1	3.8	4	6.6
Parent training	1	1.0	0	0.0	0	0.0	1	1.6
Learning therapy/treatment for dyslexia or dyscalculia/attention training	7	7.1	0	0.0	3	11.5	4	6.5
Neurofeedback	4	4.1	0	0.0	1	3.8	3	4.8
Ergotherapy	16	16.3	3	30.0	3	11.5	10	16.1
Speech therapy	6	6.1	1	10.0	2	7.7	3	4.8
Specific measures or programs at school	9	9.2	1	10.0	2	7.7	6	9.7
Physiotherapy	1	1.0	0	0.0	0	0.0	1	1.6
Youth welfare office	1	1.0	0	0.0	0	0.0	1	1.6
Others	4	4.1	0	0.0	1	3.8	3	4.8
At least one additional treatment	67	69.1	6	60.0	15	57.7	46	75.4

Note. TASH = telephone-assisted self-help, WCG = waitlist control group, BT = behavior therapy, MED = medication, Couns. = counseling; n = number of cases, χ^2 = empirical χ^2 value, df = degrees of freedom, p = significance value.

* TASH: n = 59, WCG: n = 59

** TASH: n = 60, WCG: n = 60

*** MED+BT/Couns.: n = 61

Table S10. Results of ANCOVAs for the group comparisons regarding the mean change from baseline (T1) to 3 months (T2): intention-to-treat sample; missing values imputed by Bayesian Stochastic regression

	TASH					WCG					TASH vs. WCG			
	n	Baseline/T1 M (SD)	3 months/ T2 M (SD)	Estimated marginal mean ΔT1-T2 (95% CI)	d_{intra} (95% CI)	n	Baseline/T1 M (SD)	3 months/ T2 M (SD)	Estimated marginal mean ΔT1-T2 (95% CI)	d_{intra} (95% CI)	Difference between estimated marginal means (95% CI)	SE	p	d_{inter} (95 % CI)
**Blinded clinician**														
DCL-ADHD Total	80	1.39 (0.40)	1.23 (0.48)	-0.16 (-0.26, -0.07)	-0.41 (-0.65, -0.17)	83	1.40 (0.40)	1.32 (0.47)	-0.08 (-0.17, 0.02)	-0.20 (-0.44, 0.04)	-0.08 (-0.22, 0.05)	0.07	0.22	-0.21 (-0.55, 0.13)
DCL-ADHD Impairment	80	1.14 (0.50)	1.00 (0.58)	-0.14 (-0.26, -0.03)	-0.29 (-0.52, -0.06)	83	1.14 (0.50)	1.14 (0.58)	0.00 (-0.12, 0.11)	-0.01 (-0.23, 0.22)	-0.14 (-0.30, 0.02)	0.08	0.09	-0.29 (-0.61, 0.04)
DCL-DBD Total	80	0.48 (0.35)	0.38 (0.29)	-0.12 (-0.17, -0.06)	-0.36 (-0.53, -0.20)	83	0.55 (0.31)	0.46 (0.31)	-0.07 (-0.12, -0.01)	-0.20 (-0.36, -0.03)	-0.05 (-0.13, 0.02)	0.04	0.17	-0.16 (-0.40, 0.07)
DCL-DBD ODD	80	0.84 (0.58)	0.69 (0.54)	-0.18 (-0.28, -0.08)	-0.32 (-0.51, -0.14)	83	0.93 (0.53)	0.76 (0.51)	-0.15 (-0.25, -0.05)	-0.27 (-0.45, -0.08)	-0.03 (-0.18, 0.11)	0.07	0.68	-0.05 (-0.32, 0.21)
DCL-DBD Impairment	80	0.41 (0.84)	0.53 (0.70)	0.01 (-0.16, 0.18)	0.01 (-0.19, 0.21)	83	0.73 (0.84)	0.78 (0.84)	0.16 (0.00, 0.33)	0.20 (0.00, 0.39)	-0.16 (-0.40, 0.08)	0.12	0.20	-0.19 (-0.47, 0.10)
**Semi-blinded clinician**														
DCL-ADHD Total	80	1.44 (0.33)	1.30 (0.45)	-0.15 (-0.24, -0.05)	-0.46 (-0.76, -0.16)	83	1.49 (0.31)	1.52 (0.49)	0.04 (-0.06, 0.13)	0.12 (-0.18, 0.42)	-0.19 (-0.32, -0.05)	0.07	0.008	-0.58 (-1.00, -0.15)
DCL-ADHD Impairment	80	1.25 (0.39)	1.04 (0.57)	-0.22 (-0.33, -0.11)	-0.49 (-0.74, -0.25)	83	1.28 (0.50)	1.24 (0.52)	-0.03 (-0.14, 0.08)	-0.06 (-0.31, 0.18)	-0.19 (-0.35, -0.04)	0.08	0.016	-0.43 (-0.78, -0.08)
DCL-DBD Total	80	0.49 (0.35)	0.41 (0.34)	-0.09 (-0.15, -0.04)	-0.29 (-0.46, -0.12)	83	0.57 (0.32)	0.51 (0.33)	-0.06 (-0.11, 0.00)	-0.17 (-0.34, 0.00)	-0.04 (-0.12, 0.04)	0.04	0.321	-0.12 (-0.36, 0.12)
DCL-DBD ODD	80	0.82 (0.54)	0.70 (0.52)	-0.15 (-0.25, -0.05)	-0.28 (-0.47, -0.09)	83	0.95 (0.51)	0.82 (0.54)	-0.10 (-0.20, 0.00)	-0.20 (-0.38, -0.01)	-0.05 (-0.19, 0.10)	0.07	0.53	-0.09 (-0.36, 0.18)
DCL-DBD Impairment	80	0.61 (0.63)	0.52 (0.64)	-0.11 (-0.25, 0.03)	-0.17 (-0.40, 0.05)	83	0.66 (0.60)	0.77 (0.71)	0.12 (-0.01, 0.26)	0.20 (-0.02, 0.42)	-0.23 (-0.43, -0.04)	0.10	0.021	-0.37 (-0.69, -0.06)
**Parents**														
SCL-ADHD Total	80	1.46 (0.48)	1.40 (0.48)	-0.10 (-0.21, 0.01)	-0.20 (-0.41, 0.01)	83	1.58 (0.53)	1.51 (0.55)	-0.04 (-0.14 0.07)	-0.08 (-0.29, 0.13)	-0.06 (-0.22, 0.09)	0.08	0.41	-0.13 (-0.43, 0.18)
SCL-ADHD Impairment	80	1.37 (0.70)	1.28 (0.56)	-0.12 (-0.23, -0.02)	-0.18 (-0.33, -0.03)	83	1.49 (0.66)	1.46 (0.64)	-0.01 (-0.11, 0.09)	-0.01 (-0.16, 0.14)	-0.12 (-0.26, 0.03)	0.07	0.12	-0.17 (-0.38, 0.04)
SCL-DBD Total	80	0.62 (0.43)	0.57 (0.48)	-0.06 (-0.14, 0.03)	-0.13 (-0.32, 0.07)	83	0.72 (0.47)	0.70 (0.52)	-0.01 (-0.09, 0.08)	-0.02 (-0.20, 0.17)	-0.05 (-0.17, 0.07)	0.06	0.42	-0.11 (-0.38, 0.16)
SCL-DBD ODD	80	1.02 (0.65)	0.99 (0.68)	-0.05 (-0.16, 0.06)	-0.07 (-0.23, 0.09)	83	1.15 (0.72)	1.16 (0.67)	0.02 (-0.09, 0.13)	0.03 (-0.13, 0.19)	-0.07 (-0.23, 0.08)	0.08	0.36	-0.10 (-0.33, 0.12)
SCL-DBD Impairment	80	0.99 (0.77)	0.83 (0.73)	-0.18 (-0.33, -0.04)	-0.24 (-0.43, -0.05)	83	1.11 (0.76)	1.25 (0.87)	0.16 (0.02, 0.31)	0.21 (0.03, 0.40)	-0.35 (-0.55, -0.15)	0.10	0.001	-0.45 (-0.72, -0.19)
CBCL Internalizing	80	8.21 (6.57)	8.83 (7.15)	0.77 (-0.35, 1.89)	0.13 (-0.06, 0.31)	83	7.33 (5.50)	8.17 (6.40)	0.69 (-0.41, 1.79)	0.11 (-0.07, 0.30)	0.08 (-1.50, 1.65)	0.80	0.92	0.01 (-0.25, 0.27)
WFIRS Functional Impairment	80	0.79 (0.43)	0.71 (0.39)	-0.09 (-0.16, -0.02)	-0.21 (-0.36, -0.05)	83	0.85 (0.47)	0.87 (0.46)	0.03 (-0.03, 0.10)	0.08 (-0.07, 0.23)	-0.13 (-0.23, -0.03)	0.05	0.010	-0.29 (-0.50, -0.07)

Table S10. Results of ANCOVAs for the group comparisons regarding the mean change from baseline (T1) to 3 months (T2): intention-to-treat sample; missing values imputed by Bayesian Stochastic regression

	TASH					WCG					TASH vs. WCG			
	n	Baseline/T1 M (SD)	3 months/ T2 M (SD)	Estimated marginal mean ΔT1-T2 (95% CI)	d_{intra} (95% CI)	n	Baseline/T1 M (SD)	3 months/ T2 M (SD)	Estimated marginal mean ΔT1-T2 (95% CI)	d_{intra} (95% CI)	Difference between estimated marginal means (95% CI)	SE	p	d_{inter} (95 % CI)
KIDSCREEN Quality of Life	80	37.75 (5.07)	38.03 (4.79)	0.16 (-0.78, 1.10)	0.03 (-0.16, 0.22)	83	38.17 (4.85)	38.17 (4.99)	0.12 (-0.81, 1.04)	0.02 (-0.16, 0.21)	0.04 (-1.27, 1.37)	0.67	0.95	0.01 (-0.26, 0.27)
FPNE Positive Parenting	80	1.89 (0.40)	1.93 (0.43)	0.04 (-0.03, 0.11)	0.12 (-0.07, 0.30)	83	1.88 (0.34)	1.88 (0.35)	-0.01 (-0.07, 0.06)	-0.02 (-0.20, 0.17)	0.05 (-0.05, 0.15)	0.05	0.32	0.13 (-0.13, 0.39)
FPNE Negative Parenting	80	1.92 (0.36)	1.77 (0.38)	-0.15 (-0.23, -0.07)	-0.42 (-0.64, -0.21)	83	1.93 (0.35)	1.92 (0.45)	-0.02 (-0.09, 0.06)	-0.05 (-0.26, 0.17)	-0.13 (-0.24, -0.03)	0.05	0.015	-0.38 (-0.68, -0.07)
PSBC Parental Self-efficacy	80	3.14 (0.43)	3.28 (0.40)	0.15 (0.07, 0.23)	0.32 (0.15, 0.49)	83	3.11 (0.50)	3.19 (0.49)	0.07 (-0.01, 0.15)	0.16 (-0.01, 0.32)	0.08 (-0.03, 0.19)	0.06	0.18	0.16 (-0.07, 0.40)

Note. The ANCOVAs controlled for baseline scores of the respective outcome variable and for study site. TASH = telephone-assisted self-help, WCG = waitlist control group, DCL-ADHD = Diagnostic Checklist for Attention-Deficit/Hyperactivity Disorder, ODD = oppositional defiant disorder, SCL-ADHD = Symptom Checklist for Attention-Deficit/Hyperactivity Disorder, SCL-DBD = Symptom Checklist for Disruptive Behavior Disorders, CBCL = Child Behavior Checklist, WFIRS = Weiss Functional Impairment Rating Scale, KIDSCREEN = German questionnaire for the assessment of health-related quality of life, FPNE = German questionnaire for the assessment of positive and negative parenting behaviors, PSBC = German version of the Problem Setting and Behavior Checklist.
n = sample size, M = mean, SD = standard deviation, CI = confidence interval, ΔT1-T2 = change from baseline to three-month assessment point; p = significance value, d_{intra} = effect size of within-group change (Cohen's d), d_{inter} = effect size of between-group difference (Cohen's d), SE = standard error.

Table S11. Results of ANCOVAs for the group comparisons regarding the mean change from baseline (T1) to 3 months (T2): per-protocol analysis

	TASH					WCG					TASH vs. WCG			
	n	Baseline/ T1 M (SD)	3 months/ T2 M (SD)	Estimated marginal mean ΔT1-T2 (95% CI)	d_{intra} (95% CI)	n	Baseline/ T1 M (SD)	3 months/ T2 M (SD)	Estimated marginal mean ΔT1-T2 (95% CI)	d_{intra} (95% CI)	Difference between estimated marginal means (95% CI)	SE	p	d_{inter} (95 % CI)
**Blinded clinician**														
DCL-ADHD Total	56	1.37 (0.39)	1.23 (0.42)	-0.15 (-0.24, -0.05)	-0.38 (-0.62, -0.14)	58	1.42 (0.40)	1.29 (0.43)	-0.13 (-0.22, -0.03)	-0.33 (-0.57, -0.09)	-0.02 (-0.15, 0.11)	0.07	0.77	-0.05 (-0.39, 0.29)
DCL-ADHD Impairment	53	1.08 (0.49)	0.98 (0.48)	-0.11 (-0.22, -0.01)	-0.24 (-0.45, -0.02)	49	1.11 (0.44)	1.09 (0.40)	-0.01 (-0.12, 0.09)	-0.03 (-0.25, 0.19)	-0.10 (-0.25, 0.05)	0.07	0.19	-0.20 (-0.51, 0.10)
DCL-DBD Total	52	0.45 (0.34)	0.37 (0.30)	-0.10 (-0.17, -0.04)	-0.32 (-0.53, -0.12)	49	0.54 (0.29)	0.49 (0.32)	-0.03 (-0.10, 0.03)	-0.11 (-0.32, 0.10)	-0.07 (-0.16, 0.03)	0.05	0.16	-0.21 (-0.51, 0.08)
DCL-DBD ODD	52	0.82 (0.55)	0.65 (0.48)	-0.18 (-0.30, -0.07)	-0.35 (-0.57, -0.13)	49	0.89 (0.48)	0.78 (0.50)	-0.09 (-0.21, 0.02)	-0.18 (-0.41, 0.05)	-0.09 (-0.25, 0.08)	0.08	0.30	-0.17 (-0.49, 0.15)
DCL-DBD Impairment	30	0.75 (0.51)	0.67 (0.67)	-0.06 (-0.24, 0.11)	-0.12 (-0.45, 0.22)	27	0.76 (0.54)	0.74 (0.46)	-0.04 (-0.22, 0.15)	-0.07 (-0.42, 0.29)	-0.03 (-0.29, 0.23)	0.13	0.84	-0.05 (-0.54, 0.44)
**Semi-blinded clinician**														
DCL-ADHD Total	70	1.43 (0.32)	1.28 (0.43)	-0.15 (-0.25, -0.06)	-0.49 (-0.80, -0.18)	72	1.51 (0.31)	1.48 (0.48)	-0.02 (-0.11, 0.08)	-0.05 (-0.36, 0.25)	-0.14 (-0.27, 0.00)	0.07	0.05	-0.44 (-0.87, 0.00)
DCL-ADHD Impairment	69	1.24 (0.35)	1.06 (0.52)	-0.17 (-0.28, -0.07)	-0.39 (-0.64, -0.15)	72	1.28 (0.50)	1.25 (0.49)	-0.03 (-0.13, 0.08)	-0.06 (-0.29, 0.18)	-0.15 (-0.30, 0.00)	0.08	0.05	-0.34 (-0.68, 0.00)
DCL-DBD Total	70	0.45 (0.33)	0.39 (0.32)	-0.08 (-0.14, -0.03)	-0.26 (-0.44, -0.09)	70	0.57 (0.33)	0.49 (0.32)	-0.06 (-0.11, 0.00)	-0.18 (-0.36, -0.01)	-0.03 (-0.11, 0.05)	0.04	0.52	-0.08 (-0.33, 0.17)
DCL-DBD ODD	70	0.76 (0.51)	0.67 (0.46)	-0.13 (-0.23, -0.03)	-0.25 (-0.44, -0.06)	70	0.92 (0.52)	0.81 (0.55)	-0.08 (-0.18, 0.02)	-0.16 (-0.35, 0.04)	-0.05 (-0.19, 0.09)	0.07	0.50	-0.09 (-0.37, 0.18)
DCL-DBD Impairment	69	0.57 (0.61)	0.53 (0.58)	-0.07 (-0.19, 0.06)	-0.11 (-0.32, 0.10)	71	0.66 (0.62)	0.72 (0.59)	0.08 (-0.05, 0.20)	0.12 (-0.08, 0.33)	-0.14 (-0.32, 0.03)	0.09	0.11	-0.23 (-0.53, 0.06)
**Parents**														
SCL-ADHD Total	66	1.46 (0.49)	1.39 (0.45)	-0.11 (-0.21, -0.01)	-0.22 (-0.41, -0.03)	68	1.63 (0.50)	1.56 (0.51)	-0.04 (-0.13, 0.06)	-0.07 (-0.26, 0.12)	-0.08 (-0.22, 0.06)	0.07	0.29	-0.15 (-0.42, 0.13)
SCL-ADHD Impairment	60	1.32 (0.67)	1.30 (0.53)	-0.07 (-0.19, 0.05)	-0.10 (-0.28, 0.07)	62	1.52 (0.65)	1.50 (0.64)	0.02 (-0.09, 0.14)	0.03 (-0.14, 0.21)	-0.09 (-0.26, 0.07)	0.08	0.28	-0.14 (-0.39, 0.11)
SCL-DBD Total	66	0.60 (0.45)	0.55 (0.40)	-0.07 (-0.14, 0.00)	-0.15 (-0.30, 0.00)	69	0.70 (0.47)	0.66 (0.44)	-0.02 (-0.09, 0.04)	-0.05 (-0.20, 0.09)	-0.04 (-0.14, 0.05)	0.05	0.38	-0.09 (-0.30, 0.12)
SCL-DBD ODD	66	1.01 (0.65)	0.96 (0.65)	-0.07 (-0.18, 0.05)	-0.10 (-0.27, 0.07)	69	1.11 (0.71)	1.10 (0.66)	0.01 (-0.11, 0.12)	0.01 (-0.15, 0.18)	-0.08 (-0.24, 0.09)	0.08	0.36	-0.11 (-0.35, 0.13)
SCL-DBD Impairment	61	0.98 (0.77)	0.89 (0.70)	-0.11 (-0.25, 0.03)	-0.14 (-0.33, 0.04)	60	1.09 (0.73)	1.23 (0.73)	0.15 (0.01, 0.29)	0.21 (0.01, 0.40)	-0.26 (-0.46, -0.06)	0.10	0.012	-0.35 (-0.62, -0.08)
CBCL Internalizing	66	7.99 (6.76)	7.93 (6.97)	0.08 (-1.10, 1.27)	0.01 (-0.18, 0.21)	70	7.54 (5.52)	8.16 (6.33)	0.48 (-0.66, 1.63)	0.08 (-0.11, 0.27)	-0.40 (-2.05, 1.25)	0.84	0.63	-0.07 (-0.34, 0.21)
WFIRS Functional Impairment	65	0.76 (0.39)	0.71 (0.37)	-0.06 (-0.13, 0.01)	-0.14 (-0.29, 0.01)	69	0.88 (0.47)	0.89 (0.45)	0.03 (-0.04, 0.09)	0.06 (-0.09, 0.21)	-0.09 (-0.18, 0.01)	0.05	0.07	-0.20 (-0.41, 0.02)
KIDSCREEN Quality of Life	64	38.02 (4.96)	37.85 (4.25)	-0.20 (-1.08, 0.68)	-0.04 (-0.22, 0.14)	65	37.95 (4.41)	37.71 (4.36)	-0.21 (-1.09, 0.66)	-0.04 (-0.22, 0.13)	0.01 (-1.23, 1.26)	0.63	0.98	0.00 (-0.25, 0.26)
FPNE Positive Parenting	66	1.88	1.94	0.06	0.16	70	1.87	1.86	-0.01	-0.03	0.07	0.04	0.09	0.19

Table S11. Results of ANCOVAs for the group comparisons regarding the mean change from baseline (T1) to 3 months (T2): per-protocol analysis

	TASH					WCG					TASH vs. WCG			
	n	Baseline/ T1 *M* (*SD*)	3 months/ T2 *M* (*SD*)	Estimated marginal mean ΔT1-T2 (95% CI)	d_{intra} (95% CI)	*n*	Baseline/ T1 *M* (*SD*)	3 months/ T2 *M* (*SD*)	Estimated marginal mean ΔT1-T2 (95% CI)	d_{intra} (95% CI)	Difference between estimated marginal means (95% CI)	*SE*	*p*	d_{inter} (95 % CI)
		(0.39)	(0.36)	(0.00, 0.12)	(0.00, 0.32)		(0.34)	(0.29)	(-0.07, 0.05)	(-0.18, 0.12)	(-0.01, 0.15)			(-0.03, 0.40)
FPNE Negative Parenting	65	1.86	1.74	-0.14	-0.40	69	1.93	1.94	0.02	0.05	-0.16	0.04	0.001	-0.45
		(0.34)	(0.32)	(-0.20, -0.08)	(-0.58, -0.22)		(0.35)	(0.37)	(-0.04, 0.08)	(-0.13, 0.23)	(-0.24, -0.07)			(-0.71, -0.20)
PSBC Parental Self-efficacy	64	3.16	3.27	0.14	0.29	68	3.07	3.14	0.05	0.11	0.08	0.06	0.15	0.18
		(0.43)	(0.40)	(0.05, 0.22)	(0.11, 0.46)		(0.49)	(0.46)	(-0.03, 0.13)	(-0.06, 0.28)	(-0.03, 0.20)			(-0.07, 0.43)
**Teachers**														
SCL-ADHD Total	33	1.52	1.33	-0.19	-0.33	37	1.40	1.37	-0.03	-0.06	-0.16	0.10	0.13	-0.28
		(0.65)	(0.65)	(-0.34, -0.04)	(-0.59, -0.08)		(0.52)	(0.60)	(-0.17, 0.11)	(-0.30, 0.19)	(-0.36, 0.05)			(-0.64, 0.08)
SCL-ADHD Impairment	27	1.75	1.57	-0.16	-0.24	29	1.82	1.80	-0.03	-0.04	-0.14	0.15	0.37	-0.20
		(0.79)	(0.73)	(-0.38, 0.05)	(-0.54, 0.07)		(0.65)	(0.61)	(-0.23, 0.18)	(-0.33, 0.25)	(-0.44, 0.16)			(-0.63, 0.24)
SCL-DBD Total	35	0.80	0.62	-0.15	-0.26	37	0.53	0.54	-0.02	-0.03	-0.13	0.09	0.14	-0.23
		(0.75)	(0.62)	(-0.27, -0.03)	(-0.47, -0.05)		(0.44)	(0.51)	(-0.14, 0.10)	(-0.24, 0.18)	(-0.31, 0.04)			(-0.54, 0.08)
SCL-DBD ODD	35	0.99	0.80	-0.16	-0.22	37	0.69	0.76	0.06	0.08	-0.22	0.12	0.06	-0.30
		(0.94)	(0.80)	(-0.33, 0.00)	(-0.45, 0.00)		(0.58)	(0.71)	(-0.10, 0.21)	(-0.14, 0.29)	(-0.45, 0.01)			(-0.62, 0.01)
SCL-DBD Impairment	23	1.54	1.24	-0.27	-0.29	28	1.35	1.30	-0.07	-0.08	-0.20	0.17	0.26	-0.21
		(1.05)	(0.92)	(-0.52, -0.02)	(-0.56, -0.02)		(0.88)	(0.82)	(-0.30, 0.15)	(-0.32, 0.16)	(-0.54, 0.15)			(-0.59, 0.16)

Note. The ANCOVAs controlled for baseline scores of the respective outcome variable and for study site. The per-protocol sample comprises participants whose parents participated in at least 7 telephone consultations. TASH = telephone-assisted self-help, WCG = waitlist control group, DCL-ADHD = Diagnostic Checklist for Attention-Deficit/Hyperactivity Disorder, DCL-DBD = Diagnostic Checklist for Disruptive Behavior Disorders, SCL-ADHD = Symptom Checklist for Attention-Deficit/Hyperactivity Disorder, SCL-DBD = Symptom Checklist for Disruptive Behavior Disorders, ODD = oppositional defiant disorder, CBCL = Child Behavior Checklist, WFIRS = Weiss Functional Impairment Rating Scale, KIDSCREEN = German questionnaire for the assessment of health-related quality of life, FPNE = German questionnaire for the assessment of positive and negative parenting behaviors, PSBC = German version of the Problem Setting and Behavior Checklist.
n = sample size, M = mean, SD = standard deviation, CI = confidence interval, ΔT1-T2 = change from baseline to three-month assessment point, p = significance value, d_{intra} = effect size of within-group change (Cohen's d), d_{inter} = effect size of between-group difference (Cohen's d), SE = standard error.

Table S12. Results of analyses of piecewise mixed-effects models for repeated measures for the changes within the full responders to TASH from T1 to T4: intention-to-treat sample

	Pre-TASH		Post-TASH/Step 2 Baseline		T3		T4		Change Pre-TASH to Post-TASH/Step 2 Baseline				Change Step 2 Baseline–T3				Change T3–T4				Change Pre-TASH to Post-TASH/Step 2 Baseline vs. Change Step 2 Baseline–T3	
	n	M (SD)	n	M (SD)	n	M (SD)	n	M (SD)	Estimated marginal mean (95% CI)	SE	p	d_{intra} (95% CI)	Estimated marginal mean (95% CI)	SE	p	d_{intra} (95% CI)	Estimated marginal mean (95% CI)	SE	p	d_{intra} (95% CI)	t	p
**Semi-blinded clinician**																						
DCL-ADHD Total	12	1.19 (0.35)	12	0.63 (0.17)	12	0.61 (0.36)	12	0.63 (0.33)	-0.19 (-0.24, -0.13)	0.02	< 0.001	-1.60 (-2.03, -1.16)	0.00 (-0.04, 0.03)	0.02	0.80	-0.07 (-0.61, 0.47)	0.01 (-0.07, 0.08)	0.04	0.87	0.05 (-0.60, 0.71)	-6.18	< 0.001
DCL-ADHD Impairment	12	1.17 (0.38)	12	0.85 (0.38)	12	0.57 (0.36)	12	0.67 (0.29)	-0.11 (-0.21, 0.00)	0.05	0.048	-0.83 (-1.66, -0.01)	-0.05 (-0.09, 0.00)	0.02	0.049	-0.75 (-1.49, 0.00)	0.03 (-0.05, 0.11)	0.04	0.40	0.26 (-0.37, 0.89)	-0.87	0.38
DCL-DBD Total	12	0.27 (0.19)	12	0.15 (0.11)	12	0.16 (0.16)	12	0.16 (0.18)	-0.04 (-0.06, -0.01)	0.01	0.005	-0.60 (-1.01, -0.19)	0.00 (-0.01, 0.01)	0.01	0.88	0.03 (-0.41, 0.48)	0.00 (-0.04, 0.04)	0.02	> 0.99	0.00 (-0.59, 0.59)	-2.70	0.007
DCL-DBD ODD	12	0.42 (0.29)	12	0.28 (0.24)	12	0.26 (0.27)	12	0.24 (0.21)	-0.05 (-0.10, 0.01)	0.03	0.10	-0.47 (-1.04, 0.10)	0.00 (-0.04, 0.03)	0.02	0.83	-0.07 (-0.76, 0.62)	-0.01 (-0.06, 0.05)	0.03	0.81	-0.07 (-0.67, 0.52)	-1.12	0.26
DCL-DBD Impairment	12	0.47 (0.45)	12	0.38 (0.49)	12	0.23 (0.25)	12	0.30 (0.30)	-0.03 (-0.16, 0.10)	0.06	0.66	-0.19 (-1.05, 0.68)	-0.03 (-0.07, 0.02)	0.02	0.27	-0.34 (-0.94, 0.27)	0.02 (-0.05, 0.09)	0.04	0.53	0.15 (-0.33, 0.63)	-0.03	0.97
**Parents**																						
SCL-ADHD Total	12	1.05 (0.45)	11	0.79 (0.29)	9	0.66 (0.27)	11	0.69 (0.31)	-0.08 (-0.17, 0.02)	0.04	0.10	-0.50 (-1.11, 0.10)	-0.02 (-0.05, 0.00)	0.01	0.06	-0.33 (-0.67, 0.01)	0.00 (-0.06, 0.06)	0.03	0.94	0.01 (-0.37, 0.40)	-1.04	0.30
SCL-ADHD Impairment	11	1.22 (0.49)	10	1.02 (0.36)	7	0.71 (0.38)	10	0.80 (0.28)	-0.06 (-0.17, 0.04)	0.05	0.24	-0.38 (-1.03, 0.27)	-0.05 (-0.10, 0.01)	0.03	0.09	-0.57 (-1.24, 0.09)	0.03 (-0.06, 0.11)	0.04	0.54	0.16 (-0.37, 0.68)	-0.22	0.82
SCL-DBD Total	12	0.36 (0.32)	11	0.21 (0.18)	9	0.20 (0.21)	11	0.20 (0.19)	-0.04 (-0.09, 0.01)	0.02	0.09	-0.39 (-0.85, 0.06)	0.00 (-0.01, 0.01)	0.01	0.80	-0.03 (-0.23, 0.18)	-0.01 (-0.03, 0.00)	0.01	0.12	-0.11 (-0.26, 0.03)	-1.57	0.12
SCL-DBD ODD	12	0.59 (0.51)	11	0.39 (0.33)	9	0.34 (0.31)	11	0.32 (0.39)	-0.07 (-0.16, 0.02)	0.04	0.13	-0.41 (-0.95, 0.13)	-0.01 (-0.03, 0.01)	0.01	0.45	-0.08 (-0.30, 0.14)	-0.01 (-0.06, 0.04)	0.02	0.64	-0.07 (-0.35, 0.22)	-1.31	0.19
SCL-DBD Impairment	9	0.69 (0.55)	10	0.38 (0.36)	8	0.33 (0.41)	8	0.43 (0.38)	-0.11 (-0.22, 0.00)	0.05	0.047	-0.60 (-1.20, -0.01)	0.00 (-0.04, 0.05)	0.02	0.90	0.03 (-0.46, 0.52)	0.01 (-0.03, 0.06)	0.02	0.59	0.07 (-0.19, 0.32)	-1.57	0.12
CBCL Internalizing	12	7.25 (7.16)	11	6.55 (6.77)	9	6.80 (7.46)	10	4.40 (3.41)	-0.20 (-0.90, 0.50)	0.34	0.56	-0.08 (-0.38, 0.21)	-0.15 (-0.68, 0.37)	0.26	0.56	-0.13 (-0.57, 0.31)	-0.80 (-2.46, 0.85)	0.80	0.33	-0.34 (-1.03, 0.35)	-0.10	0.92
WFIRS Functional Impairment	12	0.64 (0.42)	11	0.43 (0.26)	9	0.38 (0.26)	11	0.36 (0.31)	-0.08 (-0.15, 0.00)	0.04	0.05	-0.53 (-1.07, 0.00)	-0.01 (-0.04, 0.01)	0.01	0.35	-0.16 (-0.51, 0.19)	0.00 (-0.04, 0.04)	0.02	0.98	0.00 (-0.30, 0.29)	-1.46	0.14
KIDSCREEN Quality of Life	12	39.25 (5.29)	11	40.09 (5.22)	7	40.43 (4.86)	10	41.40 (5.50)	0.50 (-0.56, 1.56)	0.51	0.34	0.28 (-0.32, 0.88)	0.30 (-0.23, 0.82)	0.25	0.26	0.33 (-0.26, 0.93)	-0.23 (-1.08, 0.62)	0.41	0.59	-0.13 (-0.61, 0.35)	0.29	0.77
FPNE Positive Parenting	12	1.93 (0.32)	11	2.03 (0.37)	9	2.00 (0.34)	11	2.04 (0.36)	0.05 (-0.01, 0.12)	0.03	0.11	0.49 (-0.12, 1.10)	-0.01 (-0.05, 0.03)	0.02	0.64	-0.17 (-0.93, 0.58)	0.01 (-0.06, 0.07)	0.03	0.85	0.06 (-0.57, 0.69)	1.24	0.22
FPNE Negative Parenting	11	1.86 (0.39)	11	1.58 (0.29)	9	1.66 (0.24)	11	1.73 (0.34)	-0.08 (-0.16, 0.00)	0.04	0.06	-0.60 (-1.24, 0.03)	0.01 (-0.01, 0.03)	0.01	0.47	0.11 (-0.20, 0.41)	0.02 (-0.02, 0.05)	0.02	0.37	0.12 (-0.15, 0.40)	-1.84	0.07
PSBC Parental Self-efficacy	12	3.23 (0.52)	11	3.50 (0.42)	9	3.51 (0.46)	11	3.57 (0.37)	0.10 (.0.01, 0.19)	0.04	0.032	0.58 (0.05, 1.11)	0.00 (-0.04, 0.04)	0.02	0.91	0.02 (-0.41, 0.46)	0.02 (-0.06, 0.10)	0.04	0.63	0.11 (-0.36, 0.57)	1.75	0.08

Note. The MMRMs controlled for baseline scores of the respective outcome variable and for study site. Pre-TASH = assessment before TASH, i.e. T1 for children in the TASH group during Step 1 and T2 for children in the waitlist control group during Step 1, Post-TASH/Step 2 Baseline = assessment after completion of TASH, i.e. T2 for children in the TASH group during Step 1 and T2b for children in the waitlist control group during Step 1, T3 = assessment point after six months of Step 2 treatment, T4 = follow-up assessment point three months after the end of Step 2 treatment, DCL-ADHD = Diagnostic Checklist for Attention-Deficit/Hyperactivity Disorder, DCL-DBD = Diagnostic Checklist for Disruptive Behavior Disorders, SCL-ADHD = Symptom Checklist for Attention-Deficit/Hyperactivity Disorder, SCL-DBD = Symptom Checklist for Disruptive Behavior Disorders, ODD = Oppositional Defiant Disorder, WFIRS = Weiss Functional Impairment Rating Scale, KIDSCREEN = German questionnaire for the assessment of health-related quality of life, FPNE = German questionnaire for the assessment of positive and negative parenting behaviors, PSBC = German version of the Problem Setting and Behavior Checklist.
n = sample size, M = mean, SD = standard deviation, CI = confidence interval, p = significance value, d_{intra} = effect size of within-group change (Cohen's d), SE = standard error, t = empirical t-value.

Table S13. Results of the analyses of piecewise mixed-effects models for repeated measures for the changes within the partial responders to TASH from T1 to T4: intention-to-treat sample

	Pre-TASH		Post-TASH/Step 2 Baseline		T3		T4		Change Pre-TASH to Post-TASH/Step 2 Baseline				Change Step 2 Baseline–T3				Change T3–T4				Change Pre-TASH to Post-TASH/Step 2 Baseline vs. Change Step 2 Baseline–T3	
	n	M (SD)	n	M (SD)	n	M (SD)	n	M (SD)	Estimated marginal mean (95% CI)	SE	p	d_{intra} (95% CI)	Estimated marginal mean (95% CI)	SE	p	d_{intra} (95% CI)	Estimated marginal mean (95% CI)	SE	p	d_{intra} (95% CI)	t	p
**Semi-blinded clinician**																						
DCL-ADHD Total	40	1.55 (0.43)	40	1.03 (0.31)	32	1.17 (0.41)	31	1.12 (0.50)	-0.17 (-0.21, -0.14)	0.02	< 0.001	-1.20 (-1.45, -0.96)	0.02 (0.00, 0.04)	0.01	0.05	0.29 (0.00, 0.59)	-0.01 (-0.06, 0.04)	0.03	0.63	-0.08 (-0.43, 0.26)	-9.38	< 0.001
DCL-ADHD Impairment	40	1.29 (0.43)	40	0.99 (0.52)	32	0.96 (0.53)	31	0.96 (0.59)	-0.10 (-0.15, -0.05)	0.03	< 0.001	-0.70 (-1.07, -0.33)	0.00 (-0.03, 0.03)	0.02	0.98	0.00 (-0.41, 0.42)	0.00 (-0.06, 0.06)	0.03	0.91	-0.02 (-0.44, 0.39)	-2.88	0.004
DCL-DBD Total	39	0.48 (0.33)	40	0.38 (0.28)	32	0.42 (0.31)	31	0.39 (0.36)	-0.04 (-0.07, 0.00)	0.02	0.027	-0.34 (-0.63, -0.04)	0.00 (-0.01, 0.02)	0.01	0.56	0.08 (-0.18, 0.33)	-0.01 (-0.05, 0.02)	0.02	0.49	-0.12 (-0.45, 0.21)	-2.15	0.032
DCL-DBD ODD	39	0.87 (0.55)	40	0.66 (0.47)	32	0.71 (0.49)	31	0.68 (0.53)	-0.07 (-0.13, -0.01)	0.03	0.022	-0.38 (-0.70, -0.06)	0.01 (-0.02, 0.04)	0.01	0.56	0.09 (-0.21, 0.38)	-0.01 (-0.07, 0.05)	0.03	0.65	-0.07 (-0.39, 0.24)	-2.11	0.035
DCL-DBD Impairment	40	0.62 (0.56)	39	0.49 (0.55)	32	0.47 (0.45)	31	0.39 (0.40)	-0.04 (-0.10, 0.02)	0.03	0.18	-0.22 (-0.54, 0.10)	0.00 (-0.03, 0.03)	0.02	0.79	-0.04 (-0.37, 0.28)	-0.03 (-0.08, 0.03)	0.03	0.37	-0.14 (-0.44, 0.17)	-0.97	0.33
**Parents**																						
SCL-ADHD Total	38	1.47 (0.50)	38	1.25 (0.43)	31	1.22 (0.51)	27	1.12 (0.49)	-0.09 (-0.14, -0.05)	0.02	< 0.001	-0.55 (-0.82, -0.27)	-0.02 (-0.04, 0.01)	0.01	0.18	-0.19 (-0.47, 0.09)	-0.04 (-0.09, 0.01)	0.03	0.10	-.0.25 (-0.56, 0.05)	-2.81	0.005
SCL-ADHD Impairment	37	1.47 (0.66)	38	1.19 (0.55)	28	1.20 (0.63)	25	1.12 (0.52)	-0.10 (-0.17, -0.04)	0.03	0.003	-0.47 (-0.77, -0.17)	0.00 (-0.03, 0.03)	0.02	0.97	-0.01 (-0.28, 0.27)	-0.03 (-0.10, 0.04)	0.04	0.37	-0.15 (-0.47, 0.17)	-2.51	0.012
SCL-DBD Total	39	0.63 (0.45)	38	0.52 (0.42)	31	0.60 (0.48)	27	0.55 (0.40)	-0.04 (-0.08, 0.00)	0.02	0.047	-0.26 (-0.52, 0.00)	0.01 (-0.01, 0.03)	0.01	0.28	0.13 (-0.11, 0.36)	-0.04 (-0.08, 0.01)	0.02	0.10	-0.24 (-0.53, 0.04)	-2.14	0.032
SCL-DBD ODD	39	1.05 (0.63)	38	0.87 (0.58)	31	0.99 (0.76)	27	0.86 (0.67)	-0.06 (-0.11, -0.02)	0.02	0.008	-0.31 (-0.54, -0.08)	0.01 (-0.02, 0.04)	0.01	0.44	0.10 (-0.16, 0.37)	-0.06 (-0.13, 0.01)	0.03	0.07	-0.29 (-0.60, 0.03)	-2.64	0.008
SCL-DBD Impairment	34	1.09 (0.81)	35	0.93 (0.69)	27	0.89 (0.79)	25	0.67 (0.63)	-0.06 (-0.13, 0.02)	0.04	0.13	-0.21 (-0.49, 0.06)	0.00 (-0.03, 0.04)	0.02	0.81	0.03 (-0.25, 0.32)	-0.09 (-0.17, -0.01)	0.04	0.024	-0.33 (-0.62, -0.04)	-1.36	0.18
CBCL Internalizing	39	7.35 (6.68)	38	7.26 (5.79)	31	6.28 (5.62)	27	5.81 (6.65)	-0.14 (-0.73, 0.46)	0.30	0.65	-0.06 (-0.33, 0.21)	-0.15 (-0.31, 0.00)	0.08	0.06	-0.14 (-0.28, 0.04)	-0.24 (-0.69, 0.21)	0.23	0.30	-0.11 (-0.31, 0.10)	0.05	0.96
WFIRS Functional Impairment	39	0.78 (0.45)	37	0.68 (0.34)	30	0.76 (0.45)	26	0.63 (0.39)	-0.05 (-0.08, -0.01)	0.02	0.006	-0.30 (-0.52, -0.09)	0.01 (-0.01, 0.03)	0.01	0.21	0.15 (-0.09, 0.38)	-0.04 (-0.08, -0.01)	0.02	0.001	-0.29 (-0.51, -0.07)	-2.70	0.007
KIDSCREEN Quality of Life	39	38.53 (5.07)	36	39.02 (3.68)	28	39.50 (4.46)	26	39.13 (4.34)	0.15 (-0.33, 0.62)	0.24	0.54	0.09 (-0.19, 0.37)	0.12 (-0.10, 0.35)	0.11	0.29	0.15 (-0.12, 0.41)	-0.09 (-0.65, 0.46)	0.28	0.74	-0.06 (-0.38, 0.27)	0.08	0.93
FPNE Positive Parenting	39	1.83 (0.34)	37	1.89 (0.37)	31	1.90 (0.33)	27	1.86 (0.35)	0.02 (-0.02, 0.05)	0.02	0.31	0.16 (-0.15, 0.47)	0.01 (-0.01, 0.03)	0.01	0.42	0.13 (-0.19, 0.46)	-0.01 (-0.04, 0.03)	0.02	0.73	-0.05 (-0.34, 0.24)	0.45	0.65
FPNE Negative Parenting	39	1.93 (0.37)	37	1.78 (0.35)	31	1.79 (0.34)	27	1.71 (0.30)	-0.05 (-0.08, -0.02)	0.02	0.003	-0.41 (-0.67, -0.14)	0.00 (-0.02, 0.02)	0.01	0.96	0.01 (-0.25, 0.27)	-0.04 (-0.06, -0.01)	0.01	0.004	-0.30 (-0.50, -0.10)	-2.35	0.019

Table S13. Results of the analyses of piecewise mixed-effects models for repeated measures for the changes within the partial responders to TASH from T1 to T4: intention-to-treat sample

	Pre-TASH		Post-TASH/Step 2 Baseline		T3		T4		Change Pre-TASH to Post-TASH/Step 2 Baseline				Change Step 2 Baseline–T3				Change T3–T4				Change Pre-TASH to Post-TASH/Step 2 Baseline vs. Change Step 2 Baseline–T3	
	n	M (SD)	n	M (SD)	n	M (SD)	n	M (SD)	Estimated marginal mean (95% CI)	SE	p	d_{intra} (95% CI)	Estimated marginal mean (95% CI)	SE	p	d_{intra} (95% CI)	Estimated marginal mean (95% CI)	SE	p	d_{intra} (95% CI)	t	p
PSBC Parental Self-efficacy	39	3.27 (0.39)	38	3.33 (0.46)	30	3.38 (0.42)	27	3.39 (0.46)	0.02 (-0.04, 0.08)	0.03	0.48	0.15 (-0.27, 0.57)	0.01 (-0.01, 0.04)	0.01	0.36	0.17 (-0.20, 0.55)	0.02 (-0.02, 0.07)	0.02	0.34	0.17 (-0.19, 0.53)	0.23	0.82
**Teachers**																						
SCL-ADHD Total	25	1.44 (0.66)	16	1.09 (0.64)	15	1.10 (0.61)	12	1.20 (0.76)	-0.16 (-0.25, -0.06)	0.05	0.001	-0.70 (-1.12, -0.29)	0.02 (-0.02, 0.05)	0.02	0.41	0.14 (-0.20, 0.47)	-0.01 (-0.06, 0.05)	0.03	0.82	-0.03 (-0.27, 0.21)	-3.24	0.001
SCL-ADHD Impairment	19	1.66 (0.68)	18	1.33 (0.77)	14	0.98 (0.54)	10	0.98 (0.71)	-0.09 (-0.19, 0.01)	0.05	0.07	-0.40 (-0.82, 0.03)	-0.06 (-0.11, 0.00)	0.03	0.033	-0.50 (-0.96, -0.04)	-0.06 (-0.19, 0.08)	0.07	0.38	-0.25 (-0.84, 0.33)	-0.51	0.61
SCL-DBD Total	24	0.76 (0.71)	18	0.57 (0.50)	15	0.43 (0.42)	13	0.42 (0.45)	-0.08 (-0.15, -0.02)	0.03	0.016	-0.35 (-0.63, -0.07)	0.01 (-0.02, 0.03)	0.01	0.69	0.04 (-0.18, 0.26)	-0.01 (-0.07, 0.04)	0.03	0.62	-0.06 (-0.31, 0.19)	-2.36	0.02
SCL-DBD ODD	24	1.02 (0.87)	18	0.72 (0.68)	15	0.57 (0.57)	13	0.54 (0.58)	-0.11 (-0.20, -0.02)	0.04	0.016	-0.39 (-0.70, -0.07)	0.00 (-0.04, 0.04)	0.02	0.95	0.01 (-0.29, 0.31)	-0.03 (-0.13, 0.06)	0.05	0.50	-0.11 (-0.44, 0.22)	-2.14	0.032
SCL-DBD Impairment	21	1.20 (1.00)	14	1.00 (0.83)	9	0.97 (0.72)	9	0.67 (0.75)	-0.06 (-0.16, 0.04)	0.05	0.20	-0.19 (-0.49, 0.11)	-0.02 (-0.08, 0.04)	0.03	0.54	-0.12 (-0.50, 0.27)	-0.09 (-0.22, 0.04)	0.06	0.17	-0.27 (-0.65, 0.12)	-0.72	0.47

Note. The MMRMs controlled for baseline scores of the respective outcome variable and for study site. Pre-TASH = assessment before TASH, i.e. T1 for children in the TASH group during Step 1 and T2 for children in the waitlist control group during Step 1, Post-TASH/Step 2 Baseline = assessment after completion of TASH, i.e. T2 for children in the TASH group during Step 1 and T2b for children in the waitlist control group during Step 1, T3 = assessment point after six months of Step 2 treatment, T4 = follow-up assessment point three months after the end of Step 2 treatment, DCL-ADHD = Diagnostic Checklist for Attention-Deficit/Hyperactivity Disorder, DCL-DBD = Diagnostic Checklist for Disruptive Behavior Disorders, SCL-ADHD = Symptom Checklist for Attention-Deficit/Hyperactivity Disorder, SCL-DBD = Symptom Checklist for Disruptive Behavior Disorders, ODD = Oppositional Defiant Disorder, WFIRS = Weiss Functional Impairment Rating Scale, KIDSCREEN = German questionnaire for the assessment of health-related quality of life, FPNE = German questionnaire for the assessment of positive and negative parenting behaviors, PSBC = German version of the Problem Setting and Behavior Checklist.
n = sample size, M = mean, SD = standard deviation, CI = confidence interval, p = significance value, d_{intra} = effect size of within-group change (Cohen's d), SE = standard error, t = empirical t-value.

Table S14. Results of the analyses of piecewise mixed-effects models for repeated measures for the changes within the partial responders to TASH from T1 to T4: per-protocol sample

	Pre-TASH		Post-TASH/Step 2 Baseline		T3		T4		Change Pre-TASH to Post-TASH/Step 2 Baseline				Change Step 2 Baseline–T3				Change T3–T4				Change Pre-TASH to Post-TASH/Step 2 Baseline vs. Change Step 2 Baseline–T3	
	n	M (SD)	n	M (SD)	n	M (SD)	n	M (SD)	Estimated marginal mean (95% CI)	SE	p	d_{intra} (95% CI)	Estimated marginal mean (95% CI)	SE	p	d_{intra} (95% CI)	Estimated marginal mean (95% CI)	SE	p	d_{intra} (95% CI)	t	p
**Semi-blinded clinician**																						
DCL-ADHD Total	17	1.69 (0.36)	17	1.03 (0.32)	17	1.11 (0.40)	17	1.14 (0.47)	-0.22 (-0.27, -0.17)	0.03	< 0.001	-1.82 (-2.23, -1.40)	0.01 (-0.02, 0.05)	0.02	0.40	0.22 (-0.31, 0.76)	0.01 (-0.06, 0.07)	0.03	0.82	0.06 (-0.49, 0.61)	-7.88	< 0.001
DCL-ADHD Impairment	17	1.33 (0.30)	17	0.94 (0.43)	17	0.84 (0.57)	17	0.84 (0.55)	-0.13 (-0.19, -0.07)	0.03	< 0.001	-1.30 (-1.90, -0.69)	-0.02 (-0.05, 0.02)	0.02	0.30	-0.35 (-1.03, 0.33)	0.00 (-0.07, 0.07)	0.03	> 0.99	0.00 (-0.66, 0.66)	-2.97	0.003
DCL-DBD Total	17	0.55 (0.38)	17	0.36 (0.28)	17	0.41 (0.31)	17	0.40 (0.43)	-0.07 (-0.11, -0.02)	0.02	0.005	-0.52 (-0.88, -0.17)	0.01 (-0.01, 0.03)	0.01	0.29	0.16 (-0.14, 0.45)	0.00 (-0.06, 0.05)	0.03	0.86	-0.04 (-0.46, 0.39)	-3.14	0.002
DCL-DBD ODD	17	0.95 (0.59)	17	0.60 (0.45)	17	0.70 (0.50)	17	0.68 (0.63)	-0.12 (-0.19, -0.04)	0.04	0.004	-0.58 (-0.97, -0.19)	0.02 (-0.02, 0.05)	0.02	0.31	0.16 (-0.15, 0.47)	0.00 (-0.09, 0.08)	0.04	0.91	-0.02 (-0.44, 0.39)	-3.07	0.002
DCL-DBD Impairment	17	0.76 (0.60)	17	0.51 (0.52)	17	0.39 (0.43)	17	0.38 (0.39)	-0.09 (-0.18, 0.01)	0.05	0.06	-0.43 (-0.89, 0.03)	-0.02 (-0.06, 0.02)	0.02	0.33	-0.20 (-0.60, 0.21)	0.00 (-0.07, 0.06)	0.03	0.91	-0.02 (-0.35, 0.31)	-1.25	0.21
**Parents**																						
SCL-ADHD Total	17	1.53 (0.45)	17	1.32 (0.37)	16	1.21 (0.37)	14	1.05 (0.47)	-0.07 (-0.15, 0.01)	0.04	0.10	-0.47 (-1.03, 0.10)	-0.02 (-0.05, 0.00)	0.01	0.09	-0.32 (-0.68, 0.05)	-0.06 (-0.13, 0.00)	0.03	0.05	-0.41 (-0.84, 0.01)	-0.99	0.32
SCL-ADHD Impairment	16	1.65 (0.74)	17	1.33 (0.60)	14	1.26 (0.57)	13	1.00 (0.39)	-0.11 (-0.23, 0.01)	0.06	0.08	-0.43 (-0.91, 0.05)	-0.01 (-0.06, 0.03)	0.02	0.56	-0.12 (-0.51, 0.28)	-0.09 (-0.18, 0.00)	0.05	0.05	-0.36 (-0.73, 0.00)	-1.37	0.17
SCL-DBD Total	17	0.67 (0.54)	17	0.53 (0.46)	16	0.56 (0.48)	14	0.47 (0.26)	-0.05 (-0.09, 0.00)	0.02	0.06	-0.25 (-0.52, 0.01)	0.01 (-0.01, 0.02)	0.01	0.34	0.09 (-0.09, 0.27)	-0.07 (-0.14, 0.00)	0.03	0.048	-0.38 (-0.76, 0.00)	-2.03	0.042
SCL-DBD ODD	17	1.12 (0.69)	17	0.88 (0.65)	16	0.93 (0.79)	14	0.74 (0.57)	-0.08 (-0.16, -0.01)	0.04	0.032	-0.36 (-0.68, -0.03)	0.01 (-0.02, 0.03)	0.01	0.47	0.08 (-0.14, 0.30)	-0.08 (-0.18, 0.02)	0.05	0.11	-0.35 (-0.79, 0.09)	-2.24	0.025
SCL-DBD Impairment	16	1.09 (0.96)	16	1.00 (0.72)	14	0.84 (0.78)	13	0.52 (0.54)	-0.05 (-0.16, 0.07)	0.06	0.40	-0.15 (-0.51, 0.21)	-0.01 (-0.06, 0.05)	0.03	0.79	-0.04 (-0.38, 0.29)	-0.16 (-0.26, -0.06)	0.05	0.002	-0.50 (-0.81, -0.20)	-0.62	0.54
CBCL Internalizing	17	7.66 (8.03)	17	7.30 (6.73)	16	6.82 (6.08)	14	6.04 (6.89)	-0.13 (-1.21, 0.94)	0.53	0.80	-0.05 (-0.45, 0.35)	-0.12 (-0.32, 0.07)	0.10	0.21	-0.09 (-0.24, 0.05)	-0.29 (-0.98, 0.40)	0.34	0.40	-0.11 (-0.36, 0.15)	-0.02	0.99
WFIRS Functional Impairment	17	0.79 (0.47)	17	0.67 (0.31)	15	0.77 (0.39)	13	0.58 (0.38)	-0.04 (-0.10, 0.02)	0.03	0.16	-0.26 (-0.62, 0.10)	0.01 (0.00, 0.03)	0.01	0.07	0.19 (-0.02, 0.39)	-0.07 (-0.13, -0.01)	0.03	0.031	-0.42 (-0.81, -0.04)	-1.76	0.08
KIDSCREEN Quality of Life	17	39.46 (5.83)	15	39.13 (4.42)	14	39.06 (4.47)	14	39.86 (4.15)	-0.13 (-1.00, 0.74)	0.43	0.76	-0.07 (-0.51, 0.38)	0.07 (-0.29, 0.42)	0.17	0.71	0.07 (-0.30, 0.43)	0.21 (-0.57, 0.98)	0.38	0.60	0.11 (-0.29, 0.50)	-0.37	0.71
FPNE Positive parenting	17	1.86 (0.40)	17	1.94 (0.44)	16	1.94 (0.31)	14	1.96 (0.35)	0.02 (-0.04, 0.08)	0.03	0.44	0.17 (-0.27, 0.62)	0.01 (-0.02, 0.03)	0.01	0.65	0.08 (-0.29, 0.46)	0.01 (-0.03, 0.05)	0.02	0.54	0.09 (-0.20, 0.38)	0.48	0.63
FPNE Negative parenting	17	1.94 (0.40)	17	1.79 (0.39)	16	1.76 (0.33)	14	1.70 (0.34)	-0.05 (-0.09, -0.01)	0.02	0.021	-0.37 (-0.68, -0.06)	-0.01 (-0.03, 0.01)	0.01	0.33	-0.15 (-0.46, 0.16)	-0.02 (-0.05, 0.02)	0.02	0.33	-0.13 (-0.40, 0.14)	-1.54	0.12
PSBC Parental self-efficacy	17	3.32 (0.40)	17	3.31 (0.54)	16	3.46 (0.41)	14	3.48 (0.47)	0.00 (-0.10, 0.10)	0.05	>0.99	0.00 (-0.74, 0.74)	0.02 (-0.01, 0.06)	0.02	0.17	0.37 (-0.17, 0.92)	0.03 (-0.03, 0.10)	0.03	0.31	0.26 (-0.25, 0.76)	-0.39	0.70

Note. The MMRMs controlled for baseline scores of the respective outcome variable and for study site. Pre-TASH = assessment before TASH, i.e. T1 for children in the TASH group during Step 1 and T2 for children in the waitlist control group during Step 1, Post-TASH/Step 2 Baseline = assessment after completion of TASH, i.e. T2 for children in the TASH group during Step 1 and T2b for children in the waitlist control group during Step 1, T3 = assessment point after six months of Step 2 treatment, T4 = follow-up assessment point three months after the end of Step 2 treatment, DCL-ADHD = Diagnostic Checklist for Attention-Deficit/Hyperactivity Disorder, DCL-DBD = Diagnostic Checklist for Disruptive Behavior Disorders, SCL-ADHD = Symptom Checklist for Attention-Deficit/Hyperactivity Disorder, SCL-DBD = Symptom Checklist for Disruptive Behavior Disorders, ODD = Oppositional Defiant Disorder, WFIRS = Weiss Functional Impairment Rating Scale, KIDSCREEN = German questionnaire for the assessment of health-related quality of life, FPNE = German questionnaire for the assessment of positive and negative parenting behaviors, PSBC = German version of the Problem Setting and Behavior Checklist.
n = sample size, M = mean, SD = standard deviation, CI = confidence interval, p = significance value, d_{intra} = effect size of within-group change (Cohen's d), SE = standard error, t = empirical t-value.

Table S15. Results of the analyses of piecewise mixed-effects models for repeated measures for the changes within the non-responders to TASH from T1 to T4: intention-to-treat sample

	Pre-TASH		Post-TASH/Step 2 Baseline		T3		T4		Change Pre-TASH to Post-TASH/Step 2 Baseline				Change Step 2 Baseline–T3				Change T3–T4				Change Pre-TASH to Post-TASH/Step 2 Baseline vs. Change Step 2 Baseline–T3	
	n	M (SD)	n	M (SD)	n	M (SD)	n	M (SD)	Estimated marginal mean (95% CI)	SE	p	d_{intra} (95% CI)	Estimated marginal mean (95% CI)	SE	p	d_{intra} (95% CI)	Estimated marginal mean (95% CI)	SE	p	d_{intra} (95% CI)	t	p
**Semi-blinded clinician**																						
DCL-ADHD Total	85	1.48 (0.36)	85	1.53 (0.33)	76	1.16 (0.50)	64	1.12 (0.46)	0.01 (-0.01, 0.04)	0.01	0.21	0.12 (-0.07, 0.31)	-0.06 (-0.08, -0.04)	0.01	< 0.001	-1.00 (-1.27, -0.73)	-0.01 (-0.05, 0.03)	0.02	0.55	-0.10 (-0.41, 0.22)	4.93	< 0.001
DCL-ADHD Impairment	84	1.24 (0.43)	85	1.17 (0.50)	76	0.96 (0.57)	64	0.92 (0.60)	-0.02 (-0.06, 0.01)	0.02	0.18	-0.16 (-0.39, 0.07)	-0.04 (-0.06, -0.01)	0.01	0.001	-0.50 (-0.80, -0.20)	-0.02 (-0.07, 0.03)	0.03	0.43	-0.14 (-0.50, 0.21)	0.57	0.57
DCL-DBD Total	85	0.49 (0.33)	84	0.45 (0.34)	76	0.39 (0.34)	64	0.45 (0.39)	-0.01 (-0.03, 0.00)	0.01	0.11	-0.13 (-0.29, 0.03)	-0.01 (-0.02, 0.00)	< 0.01	0.13	-0.13 (-0.30, 0.04)	0.02 (0.00, 0.04)	0.01	0.07	0.18 (-0.02, 0.37)	-0.63	0.53
DCL-DBD ODD	85	0.79 (0.53)	84	0.77 (0.50)	76	0.63 (0.54)	64	0.71 (0.56)	-0.01 (-0.04, 0.02)	0.01	0.53	-0.05 (-0.22, 0.11)	-0.02 (-0.04, 0.00)	0.01	0.016	-0.23 (-0.42, -0.04)	0.03 (-0.01, 0.06)	0.02	0.11	0.17 (-0.04, 0.38)	0.57	0.57
DCL-DBD Impairment	85	0.65 (0.64)	85	0.61 (0.61)	76	0.52 (0.58)	64	0.59 (0.63)	-0.01 (-0.06, 0.03)	0.02	0.57	-0.06 (-0.26, 0.14)	-0.01 (-0.04, 0.01)	0.01	0.20	-0.13 (-0.34, 0.07)	0.02 (-0.02, 0.07)	0.02	0.33	0.11 (-0.11, 0.34)	0.05	0.96
**Parents**																						
SCL-ADHD Total	84	1.61 (0.47)	79	1.52 (0.43)	66	1.24 (0.43)	55	1.13 (0.42)	-0.03 (-0.06, 0.00)	0.02	0.09	-0.18 (-0.38, 0.03)	-0.05 (-0.06, -0.03)	0.01	< 0.001	-0.62 (-0.83, -0.40)	-0.03 (-0.06, 0.01)	0.02	0.11	-0.19 (-0.42, 0.04)	1.01	0.31
SCL-ADHD Impairment	80	1.40 (0.65)	75	1.42 (0.60)	63	1.08 (0.57)	52	1.00 (0.60)	0.00 (-0.04, 0.04)	0.02	0.93	0.01 (-0.19, 0.21)	-0.06 (-0.08, -0.03)	0.01	< 0.001	-0.52 (-0.73, -0.32)	-0.02 (-0.07, 0.04)	0.03	0.52	-0.08 (-0.33, 0.17)	2.07	0.038
SCL-DBD Total	84	0.67 (0.45)	79	0.62 (0.43)	66	0.50 (0.36)	55	0.48 (0.37)	-0.02 (-0.04, 0.01)	0.01	0.19	-0.11 (-0.27, 0.05)	-0.02 (-0.03, -0.01)	0.01	< 0.001	-0.28 (-0.43, -0.13)	-0.01 (-0.03, 0.01)	0.01	0.43	-0.05 (-0.18, 0.08)	0.31	0.76
SCL-DBD ODD	84	1.10 (0.67)	79	1.06 (0.65)	66	0.86 (0.65)	55	0.82 (0.58)	-0.01 (-0.05, 0.02)	0.02	0.46	-0.06 (-0.23, 0.10)	-0.03 (-0.05, -0.01)	0.01	0.001	-0.29 (-0.46, -0.12)	-0.02 (-0.06, 0.01)	0.02	0.21	-0.11 (-0.27, 0.06)	0.75	0.45
SCL-DBD Impairment	82	1.13 (0.77)	78	0.95 (0.73)	62	0.77 (0.64)	54	0.81 (0.75)	-0.06 (-0.11, 0.00)	0.03	0.035	-0.23 (-0.45, -0.02)	-0.03 (-0.05, 0.00)	0.01	0.048	-0.21 (-0.43, 0.00)	0.01 (-0.04, 0.07)	0.03	0.63	0.05 (-0.17, 0.28)	-0.89	0.38
CBCL Internalizing	84	8.54 (6.35)	78	7.54 (6.05)	66	7.70 (7.24)	55	6.89 (6.30)	-0.35 (-0.69, -0.01)	0.17	0.042	-0.17 (-0.33, -0.01)	0.07 (-0.12, 0.27)	0.10	0.45	0.07 (-0.11, 0.25)	-0.19 (-0.67, 0.28)	0.24	0.43	-0.09 (-0.31, 0.13)	-1.88	0.06
WFIRS Functional Impairment	83	0.86 (0.42)	78	0.77 (0.39)	64	0.70 (0.38)	55	0.64 (0.37)	-0.02 (-0.04, 0.00)	0.01	0.06	-0.15 (-0.30, 0.00)	-0.02 (-0.03. -0.01)	0.01	0.003	-0.25 (-0.42, -0.09)	-0.01 (-0.04. 0.01)	0.01	0.29	-0.09 (-0.27, 0.08)	-0.23	0.82
KIDSCREEN Quality of Life	84	37.49 (4.45)	76	37.80 (3.83)	65	39.32 (3.97)	52	39.31 (4.28)	0.13 (-0.13, 0.39)	0.13	0.34	0.09 (-0.09, 0.26)	0.23 (0.09, 0.37)	0.07	0.002	0.31 (0.12, 0.50)	-0.09 (-0.44, 0.25)	0.18	0.59	-0.06 (-0.30, 0.17)	-0.62	0.53
FPNE Positive Parenting	84	1.90 (0.34)	78	1.91 (0.34)	66	1.92 (0.38)	53	1.90 (0.40)	0.01 (-0.01, 0.02)	0.01	0.57	0.04 (-0.11, 0.20)	0.00 (-0.01, 0.02)	0.01	0.68	0.05 (-0.18, 0.27)	-0.01 (-0.04, 0.02)	0.02	0.70	-0.05 (-0.31, 0.21)	0.18	0.85

Table S15. Results of the analyses of piecewise mixed-effects models for repeated measures for the changes within the non-responders to TASH from T1 to T4: intention-to-treat sample

	Pre-TASH		Post-TASH/Step 2 Baseline		T3		T4		Change Pre-TASH to Post-TASH/Step 2 Baseline				Change Step 2 Baseline–T3				Change T3–T4				Change Pre-TASH to Post-TASH/Step 2 Baseline vs. Change Step 2 Baseline–T3	
	n	M (SD)	n	M (SD)	n	M (SD)	n	M (SD)	Estimated marginal mean (95% CI)	SE	p	d_{intra} (95% CI)	Estimated marginal mean (95% CI)	SE	p	d_{intra} (95% CI)	Estimated marginal mean (95% CI)	SE	p	d_{intra} (95% CI)	t	p
FPNE Negative Parenting	84	1.90 (0.34)	79	1.79 (0.33)	66	1.77 (0.36)	53	1.71 (0.39)	-0.04 (-0.06, -0.02)	0.01	0.001	-0.33 (-0.54, -0.13)	-0.01 (-0.02, 0.01)	0.01	0.32	-0.10 (-0.30, 0.10)	-0.02 (-0.04, 0.01)	0.01	0.17	-0.15 (-0.37, 0.06)	-2.23	0.026
PSBC Parental Self-efficacy	83	3.07 (0.42)	78	3.23 (0.37)	64	3.33 (0.47)	51	3.32 (0.41)	0.05 (0.03, 0.08)	0.01	< 0.001	0.37 (0.19, 0.55)	0.02 (0.00, 0.03)	0.01	0.03	0.25 (0.03, 0.47)	-0.01 (-0.04, 0.02)	0.02	0.51	-0.08 (-0.32, 0.16)	2.10	0.036
**Teachers**																						
SCL-ADHD Total	50	1.43 (0.62)	41	1.26 (0.63)	28	1.07 (0.61)	24	0.88 (0.52)	-0.06 (-0.10, -0.01)	0.02	0.014	-0.28 (-0.51, -0.06)	-0.04 (-0.07, -0.02)	0.01	0.002	-0.43 (-0.70, -0.17)	-0.06 (-0.11, -0.01)	0.03	0.029	-0.27 (-0.52, -0.03)	-0.44	0.66
SCL-ADHD Impairment	42	1.71 (0.68)	38	1.36 (0.74)	23	1.23 (0.70)	20	1.01 (0.66)	-0.10 (-0.17, -0.02)	0.04	0.013	-0.43 (-0.76, -0.09)	-0.04 (-0.09, 0.02)	0.03	0.17	-0.31 (-0.75, 0.13)	-0.04 (-0.14, 0.06)	0.05	0.46	-0.16 (-0.60, 0.27)	-1.19	0.23
SCL-DBD Total	50	0.61 (0.60)	42	0.52 (0.59)	29	0.44 (0.41)	24	0.38 (0.40)	-0.04 (-0.09, 0.01)	0.02	0.09	-0.20 (-0.43, 0.03)	-0.01 (-0.04, 0.01)	0.01	0.24	-0.13 (-0.36, 0.09)	0.00 (-0.03, 0.02)	0.01	0.81	-0.02 (-0.15, 0.12)	-0.91	0.36
SCL-DBD ODD	50	0.78 (0.78)	42	0.68 (0.76)	29	0.58 (0.60)	24	0.45 (0.51)	-0.04 (-0.10, 0.01)	0.03	0.13	-0.17 (-0.39, 0.05)	-0.02 (-0.05, 0.01)	0.01	0.18	-0.14 (-0.35, 0.07)	-0.02 (-0.07, 0.03)	0.02	0.43	-0.07 (-0.26, 0.11)	-0.74	0.46
SCL-DBD Impairment	41	1.37 (0.92)	35	0.86 (0.86)	22	0.90 (0.82)	16	0.77 (0.79)	-0.19 (-0.28, -0.10)	0.04	< 0.001	-0.62 (-0.91, -0.32)	-0.01 (-0.06, 0.03)	0.02	0.57	-0.09 (-0.41, 0.23)	-0.03 (-0.14, 0.08)	0.06	0.61	-0.09 (-0.46, 0.28)	-3.01	0.003

Note. The MMRMs controlled for baseline scores of the respective outcome variable and for study site. Pre-TASH = assessment before TASH, i.e. T1 for children in the TASH group during Step 1 and T2 for children in the waitlist control group during Step 1, Post-TASH/Step 2 Baseline = assessment after completion of TASH, i.e. T2 for children in the TASH group during Step 1 and T2b for children in the waitlist control group during Step 1, T3 = assessment point after six months of Step 2 treatment, T4 = follow-up assessment point three months after the end of Step 2 treatment, DCL-ADHD = Diagnostic Checklist for Attention-Deficit/Hyperactivity Disorder, DCL-DBD = Diagnostic Checklist for Disruptive Behavior Disorders, SCL-ADHD = Symptom Checklist for Attention-Deficit/Hyperactivity Disorder, SCL-DBD = Symptom Checklist for Disruptive Behavior Disorders, ODD = Oppositional Defiant Disorder, WFIRS = Weiss Functional Impairment Rating Scale, KIDSCREEN = German questionnaire for the assessment of health-related quality of life, FPNE = German questionnaire for the assessment of positive and negative parenting behaviors, PSBC = German version of the Problem Setting and Behavior Checklist.

n = sample size, M = mean, SD = standard deviation, CI = confidence interval, p = significance value, d_{intra} = effect size of within-group change (Cohen's d), SE = standard error, t = empirical t-value.

Table S16. Parental satisfaction with the interventions

	Step 1								
	TASH			WCG (TASH after 3-month waiting period)			TASH and WCG combined		
Item	n	M (SD)	Range	n	M (SD)	Range	n	M (SD)	Range
Overall satisfaction	74	3.77 (0.88)	1–5	64	3.39 (0.99)	1–5	138	3.59 (0.95)	1–5
Satisfaction with improvement of concentration	74	3.15 (0.93)	1–5	64	2.95 (0.92)	1–5	138	3.06 (0.93)	1–5
Satisfaction with improvement of other problems	74	3.35 (0.91)	1–5	64	3.23 (0.99)	1–5	138	3.30 (0.95)	1–5
Satisfaction with improvement of impairment	74	3.28 (0.80)	1–5	64	3.20 (0.93)	1–5	138	3.25 (0.86)	1–5
Satisfaction with improvement of distress	73	3.32 (0.83)	1–5	64	3.20 (0.95)	1–5	137	3.26 (0.89)	1–5
Satisfaction with improvement of distress in family	74	3.68 (0.98)	1–5	64	3.44 (1.13)	1–5	138	3.57 (1.05)	1–5
Satisfaction with amount of help received	74	3.26 (1.02)	1–5	64	3.13 (1.12)	1–5	138	3.20 (1.07)	1–5
Dealing more effectively with problems	74	3.95 (0.95)	1–5	64	3.61 (1.14)	1–5	138	3.79 (1.05)	1–5
Total score	73	3.46 (0.75)	1.00–4.63	64	3.27 (0.91)	1.00–5.00	137	3.37 (0.83)	1.00–5.00
	Step 2								
	Booster TASH			Behavior Therapy			Medication plus Behavior Therapy/Counselling[a]		
Item	n	M (SD)	Range	n	M (SD)	Range	n	M (SD)	Range
Overall satisfaction	12	3.92 (0.90)	2–5	33	3.58 (0.94)	1–5	71	3.37 (1.02)	1–5
Satisfaction with improvement of concentration	12	3.83 (0.84)	2–5	33	3.09 (0.91)	1–5	70	3.11 (1.08)	1–5
Satisfaction with improvement of other problems	12	3.92 (0.79)	2–5	33	3.61 (0.90)	1–5	70	3.19 (1.04)	1–5
Satisfaction with improvement of impairment	12	3.92 (0.79)	2–5	33	3.48 (0.91)	1–5	70	3.20 (1.04)	1–5
Satisfaction with improvement of distress	12	3.92 (0.79)	2–5	33	3.58 (0.87)	1–5	70	3.14 (1.03)	1–5
Satisfaction with improvement of distress in family	12	4.00 (0.85)	2–5	33	3.55 (0.97)	1–5	70	3.16 (1.16)	1–5
Satisfaction with amount of help received	12	3.83 (0.72)	2–5	33	3.61 (0.97)	1–5	70	3.11 (1.20)	1–5
Dealing more effectively with problems	12	4.42 (0.67)	3–5	33	3.67 (1.05)	1–5	70	3.40 (1.01)	1–5
Total score	12	3.97 (0.68)	2.13–4.63	33	3.52 (0.78)	1.00 – 4.75	70	3.21 (0.93)	1.00 – 5.00

Note. TASH = telephone-assisted self-help, WCG = waitlist control group; n = sample size, M = mean (items rated on a five-point Likert scale ranging from 1 to 5); SD = standard deviation.

[a] To our knowledge, only 32 children in this group received medication (information on medication status available for 48 children).

Table S17. Psychological side effects

	TASH	TASH after waiting period	Booster TASH	BT	MED+BT/ Couns.
	n/n_{total} (%)	n/n_{total} (%)	n/n_{total} (%)	n/n_{total} (%)	n/n_{total} (%)
**Caregivers**					
Lack of leisure time or limited contacts with peers	7/73 (9.6)	3/48 (6.3)	2/12 (16.7)	5/32 (15.6)	10/75 (13.3)
Increased conflicts	8/73 (11.0)	1/48 (2.1)	1/12 (8.3)	3/32 (9.4)	7/75 (9.3)
Fear that others will find out about therapy	3/73 (4.1)	1/48 (2.1)	1/12 (8.3)	1/32 (3.1)	4/75 (5.3)
Feeling of change for the negative	1/73 (1.4)	1/48 (2.1)	0/12 (0.0)	0/32 (0.0)	1/74 (1.4)
Irritable or bad mood	2/73 (2.7)	1/48 (2.1)	0/12 (0.0)	3/32 (9.4)	4/75 (5.3)
Time or organizational burden	3/55 (5.5)	1/41 (2.4)	0/12 (0.0)	4/28 (14.3)	1/57 (1.8)
Others	1/55 (1.8)	0/41 (0.0)	0/12 (0.0)	1/28 (3.6)	0/57 (0.0)
**Children**					
Lack of leisure time time or limited contacts with peers	1/72 (1.4)	2/48 (4.2)	0/12 (0.0)	8/32 (25.0)	13/75 (17.3)
Increased conflicts	6/72 (8.3)	2/48 (4.2)	0/12 (0.0)	3/32 (9.4)	8/75 (10.7)
Fear that others will find out about therapy	6/72 (8.3)	0/48 (0.0)	0/12 (0.0)	0/32 (0.0)	6/75 (8.0)
Feeling of change for the negative	0/72 (0.0)	1/48 (2.1)	0/12 (0.0)	0/32 (0.0)	3/74 (4.1)
Irritable or bad mood	2/72 (2.8)	1/48 (2.1)	0/12 (0.0)	7/32 (21.9)	8/75 (10.7)
Time or organizational burden	0/53 (0.0)	1/41 (2.4)	0/6 (0.0)	0/28 (0.0)	0/57 (0.0)

Note. TASH = telephone-assisted self-help, BT = behavior therapy, MED+BT/Couns. = medication plus behavior therapy/counseling; n = number of cases with psychological side effects; n_{total} = number of cases with available data.

Table S18. CONSORT 2025 checklist of information to include when reporting a randomised trial

Section/topic	No	CONSORT 2025 checklist item description	Reported on page no.
**Title and abstract**			
Title and structured abstract	1a	Identification as a randomised trial	Title page
	1b	Structured summary of the trial design, methods, results, and conclusions	4
**Open science**			
Trial registration	2	Name of trial registry, identifying number (with URL) and date of registration	4, 9
Protocol and statistical analysis plan	3	Where the trial protocol and statistical analysis plan can be accessed	9, 31
Data sharing	4	Where and how the individual de-identified participant data (including data dictionary), statistical code and any other materials can be accessed	31
Funding and conflicts of interest	5a	Sources of funding and other support (eg, supply of drugs), and role of funders in the design, conduct, analysis and reporting of the trial	33
	5b	Financial and other conflicts of interest of the manuscript authors	31–33
**Introduction**			
Background and rationale	6	Scientific background and rationale	6–9
Objectives	7	Specific objectives related to benefits and harms	8, 9
**Methods**			
Patient and public involvement	8	Details of patient or public involvement in the design, conduct and reporting of the trial	N/A
Trial design	9	Description of trial design including type of trial (eg, parallel group, crossover), allocation ratio, and framework (eg, superiority, equivalence, non-inferiority, exploratory)	9, 10, Figure 1
Changes to trial protocol	10	Important changes to the trial after it commenced including any outcomes or analyses that were not prespecified, with reason	Table S1, 12/13
Trial setting	11	Settings (eg, community, hospital) and locations (eg, countries, sites) where the trial was conducted	9
Eligibility criteria	12a	Eligibility criteria for participants	9, Table 1
	12b	If applicable, eligibility criteria for sites and for individuals delivering the interventions (eg, surgeons, physiotherapists)	N/A
Intervention and comparator	13	Intervention and comparator with sufficient details to allow replication. If relevant, where additional materials describing the intervention and comparator (eg, intervention manual) can be accessed	12, 13, Tables S2–S4

			Reported on page no.
Outcomes	14	Prespecified primary and secondary outcomes, including the specific measurement variable (eg, systolic blood pressure), analysis metric (eg, change from baseline, final value, time to event), method of aggregation (eg, median, proportion), and time point for each outcome	13, 14, Table S5
Harms	15	How harms were defined and assessed (eg, systematically, non-systematically)	14/15, Table S17
Sample size	16a	How sample size was determined, including all assumptions supporting the sample size calculation	10
	16b	Explanation of any interim analyses and stopping guidelines	N/A
Randomisation:			
Sequence generation	17a	Who generated the random allocation sequence and the method used	11
	17b	Type of randomisation and details of any restriction (eg, stratification, blocking and block size)	11
Allocation concealment mechanism	18	Mechanism used to implement the random allocation sequence (eg, central computer/telephone; sequentially numbered, opaque, sealed containers), describing any steps to conceal the sequence until interventions were assigned	11
Implementation	19	Whether the personnel who enrolled and those who assigned participants to the interventions had access to the random allocation sequence	11
Blinding	20a	Who was blinded after assignment to interventions (eg, participants, care providers, outcome assessors, data analysts)	11
	20b	If blinded, how blinding was achieved and description of the similarity of interventions	11
Statistical methods	21a	Statistical methods used to compare groups for primary and secondary outcomes, including harms	15/ 16
	21b	Definition of who is included in each analysis (eg, all randomised participants), and in which group	15/16, Table 2, Tables S10–S15
	21c	How missing data were handled in the analysis	16
	21d	Methods for any additional analyses (eg, subgroup and sensitivity analyses), distinguishing prespecified from post hoc	15/16, Tables S10, S11, S14
**Results**			
Participant flow, including flow diagram	22a	For each group, the numbers of participants who were randomly assigned, received intended intervention, and were analysed for the primary outcome	17, Figure 2
	22b	For each group, losses and exclusions after randomisation, together with reasons	17, Figure 2
Recruitment	23a	Dates defining the periods of recruitment and follow-up for outcomes of benefits and harms	10
	23b	If relevant, why the trial ended or was stopped	N/A
Intervention and comparator delivery	24a	Intervention and comparator as they were actually administered (eg, where appropriate, who delivered the intervention/comparator, how participants adhered, whether they were delivered as intended (fidelity))	12/13
	24b	Concomitant care received during the trial for each group	18, Tables S8 and S9

Baseline data	25	A table showing baseline demographic and clinical characteristics for each group	Tables S6 and S7
Numbers analysed, outcomes and estimation	26	For each primary and secondary outcome, by group:<br>● the number of participants included in the analysis<br>● the number of participants with available data at the outcome time point<br>● result for each group, and the estimated effect size and its precision (such as 95% confidence interval)<br>● for binary outcomes, presentation of both absolute and relative effect size	19-22, Table 2, Tables S12, S13 and S15
Harms	27	All harms or unintended events in each group	23, Table S17
Ancillary analyses	28	Any other analyses performed, including subgroup and sensitivity analyses, distinguishing pre-specified from post hoc	19/20, 22/23, Table S16
**Discussion**			
Interpretation	29	Interpretation consistent with results, balancing benefits and harms, and considering other relevant evidence	23–30
Limitations	30	Trial limitations, addressing sources of potential bias, imprecision, generalisability, and, if relevant, multiplicity of analyses	27/28

Citation: Hopewell S, Chan AW, Collins GS, Hróbjartsson A, Moher D, Schulz KF, et al. CONSORT 2025 Statement: updated guideline for reporting randomised trials. BMJ. 2025; 388:e081123.
https://dx.doi.org/10.1136/bmj-2024-081123
© 2025 Hopewell et al. This is an Open Access article distributed under the terms of the Creative Commons Attribution License (https://creativecommons.org/licenses/by/4.0/), which permits unrestricted use, distribution, and reproduction in any medium, provided the original work is properly cited.
*We strongly recommend reading this statement in conjunction with the CONSORT 2025 Explanation and Elaboration and/or the CONSORT 2025 Expanded Checklist for important clarifications on all the items. We also recommend reading relevant CONSORT extensions. See www.consort-spirit.org.

References

Abikoff, H., Gallagher, R., Wells, K. C., Murray, D. W., Huang, L., Lu, F., & Petkova, E. (2013). Remediating organizational functioning in children with ADHD: Immediate and long-term effects from a randomized controlled trial. *Journal of Consulting and Clinical Psychology*, *81*, 113–128.

Achenbach, T. M., & Rescorla, L. A. (2001). *Manual for the ASEBA school-age forms and profiles*. Burlington, VT: University of Vermont, Research Center for Children, Youth and Families.

Bender, R., & Lange, S. (2001). Adjusting for multiple testing—when and how?. *Journal of Clinical Epidemiology*, *54*, 343–349.

Braun, S., & Döpfner, M. (2024). *Therapieprogramm zur Steigerung von Organisationsfähigkeit, Konzentration und Impulskontrolle bei Kindern mit ADHS (THOKI-ADHS). [Therapy Program for Enhancement of Organizations Skills, Concentration and Impulse Control in Children with ADHD]*. Göttingen, Germany: Hogrefe.

Cohen, J. (1988). *Statistical power analysis for the behavioral sciences.* Hillsdale, London: Lawrence Erlbaum Associates.

Döpfner, M., Breuer, D., Schürmann, S., Wolff Metternich, T., Rademacher, C., & Lehmkuhl, G. (2004). Effectiveness of an adaptive multimodal treatment in children with Attention–Deficit Hyperactivity Disorder–global outcome. *European Child & Adolescent Psychiatry*, *13*(Suppl 1), i117-i129.

Döpfner & Görtz-Dorten (2017). *Diagnostik-System für Psychische Störungen nach ICD-10 und DSM-5 für Kinder und Jugendliche – III [Diagnostic System for Mental Disorders in Childhood and Adolescence According to ICD-10 and DSM-5].* Göttingen: Hogrefe.

Döpfner, M., Ise, E., Wolff Metternich-Kaizman, T., Schürmann, S., Rademacher, C., & Breuer, D. (2015). Adaptive multimodal treatment for children with attention-deficit-/hyperactivity disorder: An 18 month follow-up. *Child Psychiatry & Human Development*, *46*, 44–56.

Döpfner, M., Liebermann-Jordanidis, H., Kinnen, C., Hallberg, N., Mokros, L., Benien, N., ... & Dose, C. (2021). Long-term effectiveness of guided self-help for parents of children with ADHD in routine care—an observational study. *Journal of Attention Disorders*, *25*, 265–274.

Döpfner, M., Mandler, J., Breuer, D., Schürmann, S., Dose, C., Walter, D., & von Wirth, E. (2021). Children with attention-deficit/hyperactivity disorder grown up: An 18-year follow-up after multimodal treatment. *Journal of Attention Disorders*, *25*, 1801–1817.

Döpfner, M., Plück, J., Kinnen, C., & Arbeitsgruppe Deutsche Child Behavior Checklist (2014). *Elternfragebogen über das Verhalten von Kindern und Jugendlichen (CBCL/6-18R). Deutschsprachige Fassung der Child Behavior Checklist for Ages 6–18 von Thomas M. Achenbach [German version of the Child Behavior Checklist for Ages 6–18 by Thomas M. Achenbach]*. Göttingen, Germany: Hogrefe.

Döpfner, M., Schürmann, S., & Frölich, J. (2019). *Therapieprogramm für Kinder mit hyperkinetischem und oppositionellem Problemverhalten [Therapy Program for Parents of Children with Hyperkinetic and Oppositional Problem Behavior]*. (6th ed.). Weinheim, Germany: Beltz.

Dose, C., Hautmann, C., Buerger, M., Schuermann, S., Woitecki, K., & Doepfner, M. (2017). Telephone-assisted self-help for parents of children with attention-deficit/hyperactivity disorder who have residual functional impairment despite methylphenidate treatment: A randomized controlled trial. *Journal of Child Psychology and Psychiatry*, *58*, 682–690. https://doi.org/10.1111/jcpp.12661

Dose, C., Hautmann, C., & Doepfner, M. (2019). Functional impairment in children with externalizing behavior disorders: psychometric properties of the Weiss functional impairment rating scale–parent report in a German clinical sample. *Journal of Attention Disorders*, *23*, 1546–1556.

Erhart, M., Doepfner, M., Ravens-Sieberer, U., & BELLA Study Group. (2008). Psychometric properties of two ADHD questionnaires: Comparing the Conners' scale and the FBB-HKS in the general population of German children and adolescents – results of the BELLA study. *European Child and Adolescent Psychiatry, 17*(Suppl. 1), 106–115.

Goertz-Dorten, A., Ise, E., Hautmann, C., Walter, D., & Doepfner, M. (2014). Psychometric properties of a German parent rating scale for oppositional defiant and conduct disorder (FBB-SSV) in clinical and community samples. *Child Psychiatry and Human Development*, *45*, 388–397.

Guy, W. (1976). *ECDEU assessment manual for psychopharmacology – revised*. Rockville (MD): U.S. Department of Health, Education, and Welfare.

Heymans, M. W., & Eekhout, I. (2019). *Applied missing data analysis with SPSS and (R)Studio*. Amsterdam, the Netherlands: Heymans and Eekhout. https://bookdown.org/mwheymans/bookmi/

Holas, V., Thöne, A. K., Dose, C., Gebauer, S., Hautmann, C., Görtz-Dorten, A., ... & Döpfner, M. (2024). Psychometric properties of the parent-rated assessment scale of positive and negative parenting behavior (FPNE) in a German sample of school-aged children. *Child and Adolescent Psychiatry and Mental Health*, *18*, 157.

Ise, E., Kierfeld, F., & Döpfner, M. (2015). One-year follow-up of guided self-help for parents of preschool children with externalizing behavior. *The Journal of Primary Prevention*, *36*, 33–40.

KIDSCREEN Group Europe (2006). *The KIDSCREEN questionnaires: Quality of life questionnaires for children and adolescents*. Lengerich: Pabst.

Kierfeld, F., Ise, E., Hanisch, C., Goertz-Dorten, A., & Doepfner, M. (2013). Effectiveness of telephone-assisted parent-administered behavioural family intervention for preschool children with externalizing problem behavior: A randomised controlled trial. *European Child and Adolescent Psychiatry, 22*, 553–565. https://doi.org/10.1007/s00787-013-0397-7

Langberg, J. M., Epstein, J. N., Becker, S. P., Girio-Herrera, E., & Vaughn, A. J. (2012). Evaluation of the homework, organization, and planning skills (HOPS) intervention for middle school students with attention deficit hyperactivity disorder as implemented by school mental health providers. *School Psychology Review*, *41*, 342–364.

Li, G., Taljaard, M., Van den Heuvel, E. R., Levine, M. A. H., Cook, D. J., Wells, G. A., ... & Thabane, L. (2017). An introduction to multiplicity issues in clinical trials: The what, why, when and how. *International Journal of Epidemiology*, *46*, 746–755.

Mokros, L., Benien, N., Mütsch, A., Kinnen, C., Schürmann, S., Wolf Metternich-Kaizman, T., … & Döpfner, M. (2015). Angeleitete Selbsthilfe für Eltern von Kindern mit Aufmerksamkeitsdefizit-/Hyperaktivitätsstörung: Konzept, Inanspruchnahme und Effekte eines bundesweiten Angebotes – eine Beobachtungsstudie [Guided self-help interventions for parents of children with ADHD - concept, referral and effectiveness in a nationwide trial. An observational study]. *Zeitschrift für Kinder- und Jugendpsychiatrie und Psychotherapie, 43,* 275–288. https://doi.org/10.1024/1422-4917/a000348

Naumann, S., Bertram, H., Kuschel, A., Heinrichs, N., Hahlweg, K., & Döpfner, M. (2010). Der Erziehungsfragebogen (EFB). *Diagnostica*, *56*,144–57.

Organization for Economic Co-operation and Development (OECD) (1999). *Classifying educational programmes: Manual for ISCED-97 implementation in OECD countries*. Paris, France: Author.

Raudenbush, S. W., & Bryk, A. S. (2002). *Hierarchical linear models: Applications and data analysis methods*. 2nd ed. Thousand Oaks, London, New Delhi: Sage.

Ravens-Sieberer, U., Erhart, M., Rajmil, L., Herdman, M., Auquier, P., Bruil, J., ... & European KIDSCREEN Group. (2010). Reliability, construct and criterion validity of the KIDSCREEN-10 score: a short measure for children and adolescents' well-being and health-related quality of life. *Quality of Life Research*, *19*, 1487–1500.

R Core Team (2022). *R: A language and environment for statistical computing.* Vienna, Austria: R Foundation for Statistical Computing.

Sanders, M. R., Markie-Dadds, C., Tully, L. A., & Bor, W. (2000). The triple P-positive parenting program: a comparison of enhanced, standard, and self-directed behavioral family intervention for parents of children with early onset conduct problems. *Journal of Consulting and Clinical Psychology*, *68*, 624–640.

Thöne, A.-K., Görtz-Dorten, A., Altenberger, P., Dose, C., Geldermann, N., Hautmann, C., … & Döpfner, M. (2020). Toward a dimensional assessment of externalizing disorders in children: Reliability and validity of a semi-structured parent interview. *Frontiers in Psychology*, *11*, 1840. https://doi.org/10.3389/fpsyg.2020.01840

Weiss, M. D., McBride, N. M., Craig, S., & Jensen, P. (2018). Conceptual review of measuring functional impairment: Findings from the Weiss Functional Impairment Rating Scale. *BMJ Evidence Based Mental Health*, *21*, 155–164.
